# Pharmacist-Driven Chondroprotection in Osteoarthritis: A Multifaceted Approach Using Patient Education, Information Visualization, and Lifestyle Integration

**DOI:** 10.3390/pharmacy13040106

**Published:** 2025-08-01

**Authors:** Eloy del Río

**Affiliations:** Independent Researcher, 11520 Cádiz, Spain; eloy.delrio@uca.es

**Keywords:** osteoarthritis, disease-modifying osteoarthritis drugs (DMOADs), chondroprotective agents, community pharmacy-based educational interventions, medication adherence, health literacy and advice-seeking, cost-effective healthcare delivery, chronotherapeutic dosing, multidisciplinary stakeholder collaboration, P4 medicine

## Abstract

Osteoarthritis (OA) remains a major contributor to pain and disability; however, the current management is largely reactive, focusing on symptoms rather than preventing irreversible cartilage loss. This review first examines the mechanistic foundations for pharmacological chondroprotection—illustrating how conventional agents, such as glucosamine sulfate and chondroitin sulfate, can potentially restore extracellular matrix (ECM) components, may attenuate catabolic enzyme activity, and might enhance joint lubrication—and explores the delivery challenges posed by avascular cartilage and synovial diffusion barriers. Subsequently, a practical “What–How–When” framework is introduced to guide community pharmacists in risk screening, DMOAD selection, chronotherapeutic dosing, safety monitoring, and lifestyle integration, as exemplified by the CHONDROMOVING infographic brochure designed for diverse health literacy levels. Building on these strategies, the P4–4P Chondroprotection Framework is proposed, integrating predictive risk profiling (physicians), preventive pharmacokinetic and chronotherapy optimization (pharmacists), personalized biomechanical interventions (physiotherapists), and participatory self-management (patients) into a unified, feedback-driven OA care model. To translate this framework into routine practice, I recommend the development of DMOAD-specific clinical guidelines, incorporation of chondroprotective chronotherapy and interprofessional collaboration into health-professional curricula, and establishment of multidisciplinary OA management pathways—supported by appropriate reimbursement structures, to support preventive, team-based management, and prioritization of large-scale randomized trials and real-world evidence studies to validate the long-term structural, functional, and quality of life benefits of synchronized DMOAD and exercise-timed interventions. This comprehensive, precision-driven paradigm aims to shift OA care from reactive palliation to true disease modification, preserving cartilage integrity and improving the quality of life for millions worldwide.

## 1. Introduction: Context and Scope

In the emerging era of a multifaceted global health polycrisis, where health challenges are increasingly interdependent, chronic noncommunicable diseases—particularly those associated with metabolic syndrome (MetS), characterized by a constellation of metabolic abnormalities including central obesity, insulin resistance, hypertension, and dyslipidemia—have evolved into primary contributors to mortality, disability, and reduced quality of life worldwide, significantly increasing the risk of atherosclerotic cardiovascular diseases (CVDs) and type II diabetes mellitus [1]. Within this complex metabolic landscape, osteoarthritis (OA) has emerged as a silent yet pervasive epidemic, affecting over 595 million people globally [2]. This condition predominantly affects weight-bearing joints—particularly the knee and hip [3]—and is a leading cause of chronic pain and disability worldwide. Direct OA-related healthcare costs, including joint replacement surgeries and long-term care, contribute to billions of dollars annually [4]. Beyond its economic impact, OA significantly reduces the quality of life, as individuals often experience persistent pain, functional impairment, and limitations in both daily and social activities. Involvement of the knee and hip joints accounts for nearly 90% of the global years lived with disability, underscoring their dominant contribution to the overall disease burden [5]. Notably, the frequent co-occurrence of OA with other conditions associated with MetS raises critical questions about their interrelationship—whether these diseases share a common pathophysiological pathway, stem from a mutual origin, or interact bidirectionally in ways that accelerate progression and complicate management [6]. This evolving understanding calls for a shift toward earlier, more comprehensive, and multidisciplinary approaches to OA prevention and management.

Recognizing the interrelated metabolic and musculoskeletal determinants described above, effective OA management requires multifaceted and coordinated strategies implemented at the community level. In light of the growing burden of this joint disease and the pressing need for earlier multidimensional interventions, pharmacists are uniquely positioned to play a transformative role in comprehensive disease management [7,8,9,10,11,12,13,14,15,16,17,18,19,20,21,22,23,24]. As accessible and trusted healthcare professionals, community pharmacists have daily opportunities to identify at-risk individuals, provide patient education, and support adherence to both pharmacological and non-pharmacological therapies. However, their potential to promote joint health and slow OA progression remains under-recognized in most models of care [20]. Leveraging the proximity of pharmacists to the community, their expertise in medication management, and their capacity for health counseling presents a critical yet underutilized strategy to enhance early intervention and empower patients in managing OA alongside its metabolic comorbidities [20,25,26]. Effective integration of these strategies at a crucial window is imperative, considering the shared metabolic pathways and intricate interrelationships that exacerbate the burden of OA and complicate its clinical management.

This review provides a critical analysis of the current slow-acting chondroprotective strategies and underscores the multifaceted role of community pharmacies in OA management. Chondroprotection is fundamental to the pharmacological management of OA, aiming to preserve articular cartilage integrity, slow disease progression, and potentially delay the need for invasive procedures [27,28,29,30,31,32,33,34,35,36,37,38,39,40,41,42,43,44,45,46], such as total knee arthroplasty (TKA) and total hip arthroplasty (THA). Despite this, OA treatment remains largely symptomatic and relies mainly on nonsteroidal anti-inflammatory drugs (NSAIDs), other prescribed analgesics (painkillers), and widespread self-medication. However, the underlying pathophysiology of OA—characterized by an imbalance between degenerative and regenerative processes—often remains unaddressed. Without a deeper understanding of the cellular and molecular mechanisms driving this imbalance, symptomatic approaches are unlikely to modify the natural course of the disease [47,48,49,50,51,52,53]. Given the persistent gap between mechanistic knowledge and real-world practice, there is a critical need for frontline healthcare providers to translate emerging evidence into accessible, patient-centered, and effective disease-modifying interventions in routine care. Pharmacists, by virtue of their accessibility and expertise, are strategically well-placed to bridge the gap between evidence-based chondroprotection and patient implementation by offering tailored counseling on the importance of preserving cartilage integrity, clarifying dosing and proper use of agents such as glucosamine sulfate and chondroitin sulfate, and vigilant monitoring for adverse drug reactions in patients often burdened with polypharmacy. By integrating patient education, dynamic information visualization tools, and lifestyle counseling into routine practice, community pharmacies can empower patients to make informed decisions, enhance medication adherence, and engage in the proactive, holistic management of OA. To date, no systematic research has been conducted on pharmacist-led holistic chondroprotective strategies of the type proposed here, representing a significant global evidence gap and a critical opportunity for targeted investigation. This study further explores how leveraging real-world evidence and multidisciplinary collaboration can optimize pharmacist-led interventions while identifying future research and implementation challenges to advance evidence-based, synergistic, and integrated pharmacologic and non-pharmacologic strategies for chondroprotection and improved patient outcomes.

**Key Points**

▪ OA affects over 595 million people worldwide, predominately in weight-bearing joints, and is a leading cause of chronic pain, disability, and healthcare expenditure.
▪ Current management remains largely reactive and symptom-focused (NSAIDs, analgesics), without addressing the underlying imbalance between cartilage degeneration and repair.
▪ Community pharmacists—trusted, accessible medication experts—are well-placed to implement early risk screening, patient education, and chondroprotective strategies.
▪ This study critically examines slow-acting chondroprotective agents and introduces the What–How–When model, the CHONDROMOVING prototype, and the P4–4P framework to guide pharmacist-led multidisciplinary OA management.

## 2. Study Design and Methodology

A structured literature review was conducted using PubMed, Embase, and the Cochrane Library, combining controlled vocabulary (MeSH/Emtree) with free-text terms such as “osteoarthritis AND pharmacist”, “DMOADs”, “chondroprotective agents”, “chronotherapy”, “chronotherapeutic dosing”, “P4 medicine”, and “patient education AND osteoarthritis” to identify peer-reviewed clinical trials, cohort and case–control studies, systematic reviews, and meta-analyses examining pharmacist-led OA care models, disease-modifying pharmacological strategies, chronotherapy principles, and patient education interventions adapted for diverse health literacy levels. Inclusion criteria prioritized studies with direct relevance to pharmacy practice and OA disease modification; exclusion criteria eliminated non-peer-reviewed reports, conference abstracts, and articles focusing solely on non-pharmacologic management. Building on this synthesis, two complementary conceptual frameworks were developed: (1) the CHONDROMOVING prototype, a one-page infographic that translates the What–How–When model—encompassing pharmacist-led risk identification, DMOAD selection, dosing optimization, safety monitoring, and patient counseling—into intuitive, health-literacy-tailored panels for community pharmacy-based OA education; and (2) the P4–4P Chondroprotection Framework, which maps the Predictive, Preventive, Personalized, and Participatory pillars of P4 medicine onto coordinated, multidisciplinary workflows involving physicians, pharmacists, physiotherapists, and patients, thereby facilitating robust real-world implementation of chondroprotective strategies. To my knowledge, no published research has yet systematically investigated pharmacist-driven comprehensive chondroprotection strategies, such as the What–How–When and P4–4P frameworks, representing a critical knowledge gap and a timely opportunity for future studies to adopt rigorous, protocol-driven methodologies that can robustly assess their efficacy, feasibility, and scalability.

## 3. Common Pharmacological Treatments and Associated Iatrogenic Risks in OA Management

OA is often characterized as *a wound that does not heal*, a phrase that encapsulates persistent immune dysregulation, chronic low-grade inflammation, a newly described inflammatory burden index (IBI), and impaired repair processes observed in this prevalent disabling disease [49,50,51,52,53,54,55,56]. Despite its high prevalence, current therapies do not cure or delay the progression of OA. The inherent limited regenerative capacity of joint cartilage further complicates efforts to achieve satisfactory treatment outcomes. Currently, OA pharmacotherapy often begins with rapid-acting “rescue” medications, including analgesics and anti-inflammatory agents such as cyclooxygenase (COX)-2-specific drugs or selective inhibitors, corticosteroids, and opioids, to provide prompt symptom control [57,58]. Targeted joint interventions may involve intermittent intra-articular administration of glucocorticoids or hyaluronic acid formulations to enhance local relief [59,60]. Additionally, serotonin–norepinephrine reuptake inhibitors (SNRIs) may be used in repeated dosing regimens to address pain and functional impairment [61]. Although these treatments provide effective pain relief, they do not address the underlying pathophysiological mechanisms or halt disease progression, and are frequently associated with adverse events. Notably, many drugs used in the current paradigm of OA management carry significant safety risks, such as prescription analgesics, NSAIDs, and COX-2 inhibitors, including FDA Box warnings, and prolonged intra-articular glucocorticoid use, which have been associated with joint damage [62,63,64,65,66,67,68,69,70,71,72,73]. Despite significant advancements in our understanding of OA, effective disease-modifying therapies remain elusive, leaving patients trapped in a cycle of chronic pain and disability [74]. In the absence of proven therapies, even unproven interventions, such as intra-articular stem cells and platelet-rich plasma (PRP) [59,60,75,76], are sometimes employed as interim measures before total joint replacement becomes necessary. Table 1 summarizes the spectrum of OA pharmacotherapies discussed here—ranging from rapid symptomatic drugs to slow-acting agents and emerging regenerative approaches—categorized by their pharmacologic classes despite heterogeneity in underlying mechanisms and therapeutic objectives.

Iatrogenic conditions—unintended side effects and/or adverse reactions resulting from medical interventions—can occur even when standard protocols are strictly followed. Recognizing and understanding these phenomena are crucial for enhancing patient safety, optimizing treatment outcomes, and mitigating the risk of adverse drug reactions [86]. For example, NSAIDs are commonly employed in OA management for their potent analgesic and anti-inflammatory effects; however, their mechanism of inhibiting COX enzymes may inadvertently trigger various adverse outcomes. Specifically, the non-selective inhibition of both COX-1 and COX-2 not only reduces inflammation but also decreases the production of protective prostaglandins that maintain gastrointestinal mucosal integrity, thereby predisposing patients to ulcers and gastrointestinal bleeding [62,63,66,69,70]. Moreover, prolonged NSAID therapy has been linked to increased cardiovascular risks—including hypertension and myocardial infarction—and may contribute to renal impairment due to reduced renal blood flow [66]. Although traditionally regarded as a first-line, safe analgesic for OA, acetaminophen (paracetamol) now faces growing scrutiny, as recent evidence questions its modest efficacy in pain relief and highlights its hepatotoxic risks, even at therapeutic doses, particularly in patients with underlying liver disease [58,66,70]. Moreover, while opioids can provide meaningful relief in cases of refractory OA pain, their use is limited by a high potential for dependence, development of tolerance, sedation, and life-threatening respiratory depression, underscoring the need for judicious prescription and rigorous patient monitoring [71,72].

Intra-articular corticosteroid injections, despite offering short-term pain relief, have been shown to reduce cartilage thickness with repeated use and may produce systemic effects such as elevated blood sugar levels—a particular concern for diabetic patients [65,67]. Similarly, intra-articular hyaluronic acid injections aimed at restoring joint lubrication have yielded inconsistent efficacy relative to placebo in clinical trials and carry a modest risk of local inflammatory reactions [66]. Of particular interest is the emerging class of biolubricant therapies designed to augment the cartilage-surface lubricant layer (Table 1). While enhanced boundary lubrication can mitigate frictional wear, excessive thickening of this layer may paradoxically intensify diffusion barriers, impeding nutrient transport to deep chondrocytes and potentially exacerbating degenerative processes rather than ameliorating them [87]. This exemplifies the broader iatrogenic principle that modifying one aspect of tissue physiology can inadvertently compromise another and highlights the imperative for integrated strategies that balance mechanical protection with metabolic support. Finally, the efficacy of PRP injections remains uncertain, with some studies indicating functional improvements without corresponding pain relief and an associated increased risk of adverse effects [76].

Drug-induced complications, overmedicalization, and treatment-related side effects remain critical contributors to iatrogenic outcomes in OA pharmacological management. Notably, many widely cited estimates of NSAID-related morbidity and mortality derive from seminal epidemiological investigations conducted over twenty years ago, thereby limiting their relevance to current clinical practice. For instance, Griffin et al. [62] reported approximately 41,000 hospitalizations and 3,300 deaths annually among older adults in the United States due to NSAID-related complications. Similarly, Tramer et al. [63] estimated that NSAID use contributed to approximately 2,000 deaths annually among patients with OA in the United Kingdom. While historically informative, these figures underscore the pressing need for updated pharmacoepidemiological data to more accurately assess the contemporary risk profile of NSAID therapy in OA populations. In parallel, preliminary clinical trials have indicated that certain NSAIDs may have deleterious effects on joint structure, potentially accelerating OA progression, though much of this evidence is largely drawn from small or older cohorts [64]. These studies suggest that NSAIDs may disrupt the homeostatic mechanisms of cartilage metabolism and compromise synovial tissue integrity, leading to impaired repair processes and increased cartilage degradation. Although these findings are based on early phase trials and require further investigation, they raise concerns that the symptomatic relief provided by NSAIDs may come at the cost of exacerbating the underlying structural deterioration of osteoarthritic joints. However, robust contemporary data quantifying these structural effects in real-world OA populations remain scarce. In addition, Salis et al. [73] recently reported that long-term NSAID use in patients with knee OA was associated with a significant increase in symptom severity—including pain, disability, and stiffness—and a markedly higher likelihood of progression to total knee replacement, despite no clear radiographic evidence of structural worsening. These observations may be attributable to the analgesic effects of NSAIDs, which can mask symptom severity and thereby delay necessary behavioral modifications and interventions that might otherwise mitigate joint overuse and subsequent deterioration [68,88,89]. Together, these concerns highlight the complex and potentially paradoxical role of NSAIDs in OA management, reinforcing the need for careful, evidence-based prescriptions informed by up-to-date safety and efficacy data.

Guided by the ethical mandate *primum non nocere*—“first, do no harm”—current best practice emphasizes conservative, risk-mitigating therapies whenever feasible. Consequently, the optimal approach for OA management aims to minimize reliance on NSAIDs, adhering to the principle that they should be used at the lowest effective dose for the shortest possible duration [90]. In addition to pharmacological interventions, broader, well-structured environmental and lifestyle modifications can play a pivotal role in modifying the natural trajectory of the disease—attenuating its progression and mitigating its long-term impact [91,92,93]. As mentioned earlier, chondroprotection, which focuses on preserving cartilage integrity, is a critical component of OA management and demands the integration of targeted pharmacologic agents, supportive non-pharmacologic therapies, and timely, personalized interventions to slow disease progression and maintain joint function [94,95]. However, the adherence to these strategies remains inconsistent. Yet paradoxically—and regrettably—chondroprotection, a cornerstone strategy for modifying the natural course of OA, remains largely aspirational rather than operational, with inconsistent implementation and frequent overshadowing by the pursuit of short-term symptomatic relief. To transcend this symptom-driven paradigm, slow-acting chondroprotective agents, such as glucosamine sulfate and chondroitin sulfate, are prescribed as cartilage-supportive compounds, although their clinical efficacy remains debatable. Given these uncertainties, treatment guidelines must be developed based on rigorous scientific evidence to ensure effective and safe interventions. A balanced strategy that integrates both pharmacological and non-pharmacological therapies with the underlying pathophysiological mechanisms of OA can optimize symptom management while minimizing the risk of iatrogenic complications.

## 4. Historical and Economic Perspectives on OA Pharmacotherapy: A Brief Overview

Pharmacological management of OA has evolved significantly in recent decades, resulting in a clear distinction between the two principal therapeutic categories based on their speed of action. Initially, treatment strategies focused primarily on the use of symptomatic rapid-acting drugs (SYRADOAs)—such as simple analgesics, NSAIDs, opioids, and corticosteroids—which provide fast relief to manage acute pain episodes [57,58]. Medications such as paracetamol, ibuprofen, diclofenac, and selective COX-2 inhibitors have traditionally served as “rescue” medications to quickly alleviate the burden of pain for millions worldwide. A paradigm shift occurred in the early 1990s, when clinical research began to emphasize long-term management strategies aimed at not only managing symptoms but also modifying the underlying joint pathology. This led to the emergence of symptomatic slow-acting drugs for osteoarthritis (SYSADOAs)—including glucosamine sulfate, chondroitin sulfate, diacerein, and other slow-acting symptomatic chondroprotective agents—and the acronym ‘SYSADOA’ quickly became the convenient reference for this drug class, now formally indexed in the latest update of the Dictionary of Rheumatology [96]. These agents operate via a gradual mechanism, modulating cartilage metabolism by inhibiting lysosomal protease synthesis, reducing the generation of reactive oxygen and nitrogen species, and countering the effects of pro-inflammatory cytokines (PICs). Importantly, the therapeutic benefits of SYSADOA are cumulative, typically requiring several weeks to months of continuous use, with effects that may persist for up to two months after discontinuation [36].

From an economic perspective, global market data further highlight the impact of SYSADOA on OA management [97,98,99,100]. Although precise epidemiological data on utilization are limited, market analyses offer compelling evidence of their extensive adoption. For example, the global glucosamine market was valued at approximately USD 1.1 billion in 2023 and is projected to grow to USD 1.7 billion by 2030, reflecting a compound annual growth rate (CAGR) of 6.8% [98]. Similarly, the chondroitin sulfate market was estimated to be USD 1.31 billion in 2023, with projections indicating a CAGR of 3.5% from 2024 to 2032 [99,100]. This expansion is predominantly observed in Europe and the Asia–Pacific region, where the convergence of an aging population, increasing OA burden, supportive regulatory landscapes, favorable reimbursement policies, and rising consumer demand have collectively fueled widespread use. However, cost-effectiveness evaluations of SYSADOAs yield inconsistent results: while some studies report incremental cost-per-quality-adjusted-life-year gains within commonly accepted thresholds, others question their long-term economic value given heterogeneous clinical outcomes [101,102]. This uncertainty is exacerbated by the predominance of over-the-counter (OTC) distribution, which shifts considerable costs to patients and raises concerns about equitable access and payer coverage [103,104]. In the United States, OTC distribution dominates, driving patient out-of-pocket spending to an estimated USD 14.9 billion on complementary approaches for painful conditions—underscoring both robust consumer demand and significant gaps in reimbursement policy [105]. Together, these market dynamics underscore the urgency of robust real-world evaluations and clearer policy guidelines to ensure that SYSADOA use is both clinically justified and economically sustainable.

In summary, the historical shift from the use of rapid-acting analgesics to the adoption of SYSADOA in OA pharmacotherapy not only marks a significant advancement in treatment philosophy—from immediate symptom control to sustained joint preservation—but also mirrors the growing economic relevance of these chondroprotective agents. SYSADOAs constitute a rapidly growing sector of the OA therapeutics market, with global glucosamine sales exceeding USD 1 billion annually [97,98], and chondroitin sulfate generating similar revenue [99,100]. These robust economic indicators suggest that millions of individuals across the globe rely on SYSADOA as a key component of their long-term joint health management strategies. Nevertheless, cost-effectiveness-focused pharmacoeconomic analyses produce divergent findings, largely attributable to heterogeneity in patient-specific phenotypes and disease severity [41,102,106]. Consequently, the integration of SYSADOA into personalized treatment regimens underscores a dual commitment: achieving gradual symptomatic improvement, while potentially attenuating the progression of structural joint deterioration. As both clinical evidence and market trends continue to evolve, SYSADOA remains central to the comprehensive management of OA, providing a balanced approach that bridges short-term relief with long-term disease modification, although its precise role and effectiveness continue to be the subject of ongoing investigation and debate.

## 5. Clarifying Terminological Ambiguity in OA Pharmacological Treatments: The Need for Standardized Nomenclature in Slow-Acting Medications

Over the past several decades, a broad spectrum of pharmacological agents has been developed and investigated for the treatment of OA aimed at alleviating symptoms and modifying disease progression [27,28,29,30,31,32,33,34,35,36,37,38,39,40,41,42,43,44,45,46], yet no “causal pill” (or “magic bullet”) has emerged to fully address the multifactorial nature of this complex condition. Traditional slow-acting disease-modifying agents for OA are specifically designed to decelerate disease progression and address chronic pain in a condition inherently characterized by a slow, progressive trajectory. Despite the proliferation of these therapies, the terminology used to describe them remains inconsistent and ambiguous, which leads to confusion among researchers, clinicians, pharmacists, and patients. Terms such as “disease-modifying osteoarthritis drugs” (DMOADs), “structure-modifying OA drugs” (SMOADs), “connective tissue structure-modifying agents” (CTSMAs), “symptomatic slow-acting drugs for osteoarthritis” (SYSADOAs), and “chondroprotective agents”, also known as “chondroprotectors”, are often used interchangeably [29,79,96], despite key differences in their intended mechanisms and clinical implications. For instance, SMOADs (or CTSMAs) inherently possess structure-modifying potential by directly influencing joint morphology, whereas a subset of DMOADs exerts their disease-modifying effects by neutralizing pro-inflammatory cytokines or matrix-degrading enzymes without inducing visible structural changes. Similarly, SYSADOAs such as glucosamine and chondroitin sulfate are typically associated with gradual symptomatic relief [107], yet they are frequently referred to as chondroprotective agents, a term implying cartilage protection despite the fact that cartilage is aneural and cannot itself be a source of OA-related pain. Instead, pain in OA arises from secondary pathological changes, such as synovial inflammation and subchondral intraosseous hypertension due to venous congestion [32,108,109,110,111]. This lack of precise terminology has significant practical consequences, complicating drug development, regulatory approval, clinical trial design, and patient communication. Ambiguous classifications may lead to unrealistic patient expectations, difficulties in pharmacist counseling, and inconsistencies in clinical decision-making. Given these challenges, it is crucial to establish a standardized nomenclature for OA pharmacotherapies that accurately reflects their mechanisms, intended effects, and clinical utility. A precise and harmonized taxonomy can foster clearer communication and dialogue among clinicians, researchers, policymakers, and industry partners (such as pharmaceutical manufacturers), thereby enabling evidence-based treatment strategies and optimizing patient outcomes.

Chondroprotection (literally “cartilage protection”) has long been a central objective in OA pharmacotherapy under the assumption that maintaining cartilage integrity would yield clinically meaningful reductions in pain and disability. Yet, accumulating evidence challenges this assumption, revealing that structural integrity alone is not a reliable predictor of symptom relief [26,32,80,108,110,111,112,113,114,115,116,117]. The relationship between cartilage degeneration and pain perception in OA is complex and multifactorial, involving not only inflammatory mediators and subchondral bone changes but also fibrous and contractile periarticular tissues, including the joint capsule, ligaments, and surrounding muscles, which contribute more directly to nociception. While healthy cartilage lacks nociceptive innervation and signaling, pathological changes in OA can introduce mechanisms by which cartilage may contribute to pain. For instance, neurovascular ingrowth through small channels at the osteochondral junction has been observed, allowing pain fibers to reach the deep cartilage layers [108]. Additionally, chondrocytes in osteoarthritic cartilage are known to produce nerve growth factor, a potent mediator of pain sensitization [115]. Despite its aneural composition, cartilage degradation may further contribute to symptoms indirectly; proteolytic byproducts—such as cartilage oligomeric matrix protein (COMP), fibronectin fragments (FN-fs), and type II collagen-derived telopeptides (e.g., Helix-II, CTX-II)—released during matrix breakdown act as damage-associated molecular patterns (DAMPs), engage toll-like receptors, and trigger proinflammatory signaling cascades [51,108,110]. These findings challenge the traditional view that cartilage is a passive player in OA pain and suggest a more nuanced interplay between structural changes and clinical symptoms.

Yet emerging evidence indicates that the relationship between cartilage degeneration and pain perception in OA is far more complex than a simple direct correlation. Indeed, variations in cartilage thickness often show only weak or inconsistent correlations with patient-reported pain, underscoring the necessity of integrating additional factors beyond structural loss to fully understand OA discomfort [112,114,116]. Although loss of cartilage is a hallmark of OA, the lack of innervation limits its direct contribution to pain. Instead, the weak association between cartilage thinning and worsening pain appears to be mediated by concomitant changes in other joint structures. Specifically, cartilage loss is frequently accompanied by synovitis and bone marrow lesions—both of which are rich in nociceptive nerve endings and are more directly implicated in pain generation [110]. These findings suggest that while chondroprotective strategies may slow structural deterioration, their impact on pain relief is likely to be limited unless they address inflammatory or subchondral alterations that contribute more directly to the symptomatology of OA.

While cross-sectional MRI studies have reported that individuals with greater cartilage loss tend to experience more severe pain [108,110,111], the relationship between cartilage degradation and OA symptomatology is likely indirect and multifactorial. As mentioned earlier, cartilage itself lacks nociceptive innervation, making it an unlikely direct source of pain. Instead, its loss often co-occurs with synovium and bone marrow lesions—structures within the diarthrodial joint that are richly innervated and capable of generating pain. In a longitudinal study analyzing MRI data from 600 knees over a 24-month period, Felson et al. [111] found that cartilage thickness loss is only weakly associated with worsening knee pain. Mediation analysis further revealed that this association was partially explained by concurrent increases in synovitis but not bone marrow lesions. These findings suggest that although cartilage preservation remains a key structural target, establishing a direct pain-relieving effect of chondroprotective therapies remains challenging, as cartilage loss itself has only a limited impact on pain progression. Therefore, relying solely on subjective assessments, such as the WOMAC pain score, may inadequately capture therapeutic benefits, underscoring the need for innovative approaches that integrate chemical biomarkers with advanced imaging technologies to more accurately evaluate both the structural and symptomatic effects of pharmacological chondroprotection in OA [118,119,120].

As previously noted, SYSADOAs generally refer to compounds such as glucosamine sulfate and chondroitin sulfate, which are used for their potential to alleviate symptoms—and, controversially, may even slow disease progression—while offering a very favorable safety profile that is endorsed by a range of European clinical guidelines, specialist society position papers, and expert consensus statements [28,36,39,40,57,121,122,123,124]. In the United States, many of these slow-acting chondroprotective agents are predominantly marketed as nutritional supplements (or nutraceuticals) rather than as regulated pharmaceuticals (or formal medicines). Consequently, they fall under a less rigorous regulatory framework of the U.S. Food and Drug Administration (FDA), meaning that they are not required to meet the same stringent standards for efficacy and safety as approved pharmaceutical drugs [69,125]. Establishing a clear mechanistic link between structural changes and clinical outcomes is imperative in the context of structure/disease-modifying anti-OA drug compounds. As noted in FDA guidance [69], *to accept structural endpoints as valid outcome measures for accelerated approval, there should be substantial confidence that an effect on the candidate structural endpoint will reliably predict an effect on the clinical outcomes of interest*. This statement highlights the regulatory demand for robust evidence that changes in imaging biomarkers, such as cartilage thickness, synovitis, and bone marrow lesions, are directly associated with significant improvements in patient symptoms. Despite the promise of chondroprotective strategies to slow structural deterioration, current research indicates that the relationship between these structural changes and clinical pain relief remains complex and only partially mediated by inflammatory or subchondral alterations [110,111]. As such, the challenge remains to demonstrate that the impact of an intervention on structural endpoints can be reliably translated into tangible clinical benefits in patients with OA.

**Figure 1:**
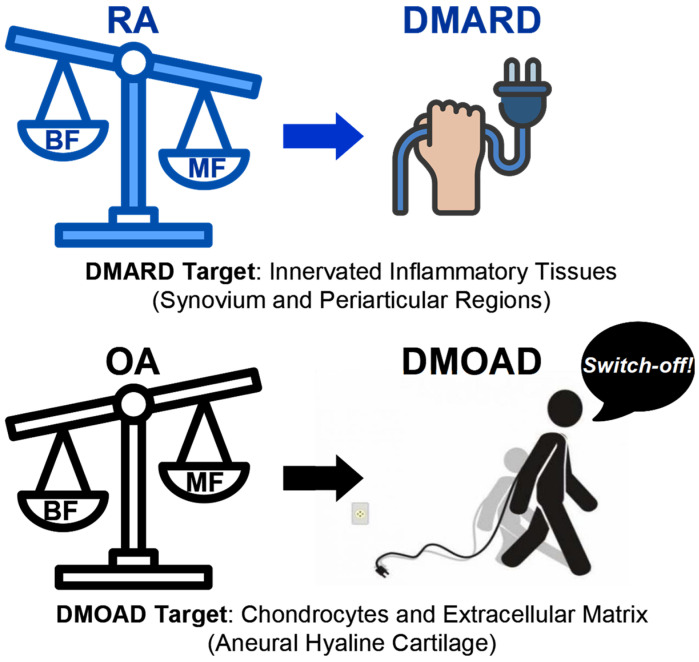
Visual analogy illustrating distinct therapeutic mechanisms in RA and OA. RA is largely driven by metabolic factors (MFs), where severe-grade, immune-mediated inflammation is the key determinant of joint damage. In OA, joint degeneration is predominantly influenced by biomechanical factors (BFs), with repetitive or cyclic loading of weight-bearing joints (e.g., knees and hips) triggering low-grade inflammatory responses and cartilage breakdown. This figure further contrasts the therapeutic strategies: DMOADs, such as glucosamine and chondroitin, target pro-catabolic factors such as ECM fragments, pro-inflammatory cytokines, and matrix-degrading enzymes in response to biomechanical stressors, whereas DMARDs, including both conventional and biological agents, address immune-driven inflammatory processes. In RA, DMARDs can be conceptualized as circuit breakers that effectively interrupt inflammatory cascades driven by metabolic factors, thereby protecting joints from immune-mediated damage. In contrast, DMOADs in OA play a role similar to that of inhibitors, attenuating the biochemical response owing to the impact of repetitive mechanical stresses that lead to cartilage degradation. The ‘unplugging’ metaphor illustrates how biomechanical factors can insidiously and confusingly contribute to disease progression, thereby limiting the ability of individuals to fully recognize or accurately interpret the therapeutic effects of chondroprotective treatment. Abbreviations: OA—osteoarthritis; RA—rheumatoid arthritis; BF—biomechanical factor; MF—metabolic factor; DMOAD—disease-modifying osteoarthritis drug; DMARD—disease-modifying anti-rheumatic drug.

Bridging the gap between cartilage preservation and genuine pain relief remains elusive—a stark reminder that established OA is a multifactorial joint disease driven by mechanical stress, metabolic dysregulation, inflammation, and subchondral bone remodeling, making its pharmacological management particularly challenging. Unlike rheumatoid arthritis (RA), in which disease-modifying antirheumatic drugs (DMARDs) can effectively suppress the autoimmune-driven inflammatory process and induce disease remission, OA lacks a comparable pharmacological approach. DMOADs primarily target cartilage metabolism in an attempt to slow degradation and promote repair. However, given that cartilage damage in OA is a downstream consequence of mechanical, inflammatory, and metabolic processes rather than a primary disease driver, these agents have failed to alter disease progression meaningfully [110,113]. In this regard, Felson and Kim argue that the deterioration of cartilage in OA is not an isolated event but rather a secondary outcome resulting from a complex interplay of factors [113]. They pointed out that mechanical stress (from abnormal or excessive loading), chronic inflammation, and altered joint biomechanics contribute to cartilage degradation. Consequently, anti-OA treatments that target only the cartilage itself—aiming simply to protect or repair it—are unlikely to succeed if the underlying mechanical, low-grade inflammatory, and metabolic disturbances within the joint environment are not targeted [113]. Only by concurrently addressing these key pathophysiological processes can chondroprotective strategies hope to achieve meaningful disease modification. As shown in Figure 1, the pathomechanical nature of OA, which is not a feature of RA, poses a fundamental obstacle to effective pharmacological intervention, since preserving cartilage alone fails to address the underlying joint-failure processes. This distinction highlights the necessity for a multimodal treatment strategy that integrates biomechanical, metabolic, pharmacological, and lifestyle-based interventions to effectively modify OA progression.

As the understanding of OA evolves, the terminology that guides its management must also evolve. Although the concept of SYSADOA once served to distinguish agents with delayed yet sustained effects, mounting evidence reveals that the relationship between structural preservation and symptomatic relief is neither linear nor consistently predictive [108,109,110,111]. This disconnect demands a critical re-examination of the classical nomenclature. Although validated biochemical biomarkers for OA remain scarce, adopting the term DMOAD provides a more precise and scientifically robust taxonomy, underscoring therapies designed to intervene in the underlying disease processes rather than merely addressing symptoms [33,41,126]. A unified lexicon will support better research alignment, regulatory clarity, and ultimately improved clinical outcomes.

## 6. Chondroprotective Pharmacotherapies for OA: Controversies, Limitations, and Challenges

Despite significant advances in therapy and ongoing research, a definitive “gold standard” treatment for OA remains elusive [46,74,78,80,81,82,83,84,85]. In this context, DMOADs have emerged as promising alternatives for preserving joint integrity and altering the course of disease progression. Notably, glucosamine and chondroitin sulfate have been classified as a subclass of DMOADs and have long been employed for their chondroprotective properties [29,33,41,79,91,106]. However, despite decades of clinical use and extensive research, these agents continue to be enveloped by substantial controversies, limitations, and challenges that impede their widespread acceptance and optimal application. Adding to these scientific and clinical challenges are regulatory and economic factors [101,103,104,105]. In many regions, glucosamine and chondroitin sulfate are classified as dietary supplements rather than pharmaceuticals, which adversely impacts the rigor of quality control, safety monitoring, and reimbursement policies. Although these chondroprotective agents are generally well tolerated, concerns regarding product variability and the potential for adverse drug interactions—especially in patients with polypharmacy—remain [39,40]. Furthermore, the economic burden of OA, driven by high direct healthcare costs, including joint replacement surgeries and long-term care, emphasizes the need for cost-effective and evidence-based interventions that can be sustainably integrated into clinical practice [4].

The rather short but intense history of the use of chondroprotective agents cannot be fully understood without examining the debate regarding its controversial efficacy [35,37,41,78,94,113,122,126,127,128,129,130,131,132,133]. Although continuous discussions have stimulated extensive research within the scientific community, the precise effectiveness of these agents remains unclear. Owing to the increasing popularity of cost-effective conventional anti-OA therapies based on glucosamine and chondroitin sulfate, there is growing interest in the widespread use of slow-acting chondroprotective agents [78]. When used in combination, glucosamine and chondroitin sulfate have been proposed as promising candidates for the treatment of OA [29,31,32,34,36,38,39,40,41,45,134]. Despite promising preclinical results, the efficacy of these DMOADs as conservative treatments for knee and hip OA remains a subject of controversy in the scientific and medical literature, particularly because of the heterogeneous effects observed in randomized, double-blind, placebo-controlled trials [127]. Indeed, effectiveness tends to be lower than safety (no interaction with human hepatic cytochrome isozymes) [135,136]. Despite increasing attention to effectiveness issues in recent years, insufficient progress has been made on this matter, which remains unresolved. This highlights the need for further research to better understand the factors contributing to their variable effects [94].

As discussed above, the role of chondroprotective agents, such as glucosamine and chondroitin sulfate, in OA management has been a subject of considerable debate, with clinical evidence presenting contrasting perspectives on their efficacy. Various factors, including differences in salt types, biases in study design, and other confounding variables, may explain some inconsistencies in the evidence [33,34,41,127,128,133,134,137,138]. Some studies suggest that these agents may offer symptomatic relief by slowing the progression of OA and improving joint function, particularly in the early stages of the disease. Clinical studies and meta-analyses have reported conflicting results regarding the therapeutic efficacy of chondroprotective agents. Variability in study design, patient selection, dosage regimens, and outcome measures has contributed to ongoing debates regarding their true clinical value in slowing disease progression or alleviating symptoms [127]. While some investigations report modest improvements in pain and joint function, others fail to demonstrate significant benefits over placebo, leaving both clinicians and patients uncertain regarding the utility of these agents in routine practice. For example, a meta-analysis conducted by Zhu et al. [34] indicated that glucosamine and chondroitin sulfate may provide moderate improvements in pain and function in knee OA patients. However, other clinical trials have failed to demonstrate significant benefits compared to placebos, leading to skepticism regarding their disease-modifying potential. A notable example is the GAIT trial [128,137,138], which found minimal improvements in pain and function with glucosamine and chondroitin sulfate in patients with knee OA, particularly in those with moderate-to-severe disease.

The current body of evidence is further complicated by methodological limitations [33,34,41]. Many studies suffer from issues related to short follow-up periods, inadequate sample sizes, and heterogeneity in diagnostic criteria. In addition, there is a notable lack of robust data linking biochemical markers of cartilage metabolism to long-term clinical outcomes. This gap highlights the need for more rigorous long-term clinical trials that can better define the patient subgroups most likely to benefit from chondroprotective therapies and elucidate the underlying mechanisms of action. In particular, focusing on early-stage OA endotypes rather than the traditional emphasis on end-stage OA phenotypes could provide more targeted insights in the context of patient stratification [106,139,140]. Despite controversial evidence, the persistence of glucosamine and chondroitin sulfate in clinical practice reflects a demand for more effective disease-modifying therapies for OA, underscoring the importance of exploring novel approaches that may complement or enhance their effects.

## 7. Silent Signals and Early Shields: Proactive Chondroprotection to Modify the Disease Trajectory of Subclinical OA

Despite its high prevalence and impact, OA is often misunderstood as a benign and inevitable aspect of aging rather than a progressive and debilitating joint disease that drives substantial morbidity, functional impairment, and increased risk of all-cause mortality [56]. This widespread misperception and persistent misconception contribute to underestimating the clinical significance of this joint condition and delay timely medical engagement, which is critical for effective nonsurgical intervention [22,23,26,141,142,143,144,145,146,147,148,149,150,151,152,153,154,155,156,157,158,159,160,161]. The insidious progression of OA coupled with variability in symptoms and disease trajectories further obscures its true burden. As a result, patients frequently seek care only in advanced stages when joint damage is severe, and therapeutic options are limited [158]. Exacerbating this issue is a systemic obstacle to timely diagnosis and treatment that disproportionately affects marginalized groups with low health literacy, limited healthcare access, and socioeconomic constraints [3,162,163,164,165,166,167]. Overcoming these challenges requires a paradigm shift in both public health messaging and clinical practice—one that redefines OA as a serious, modifiable condition warranting early, patient-centered, and multidisciplinary management supported by proactive, evidence-based, and mechanism-informed treatment strategies, where data-driven insights meet innovative frameworks to reconceptualize joint disease as a modifiable continuum.

**Figure 2:**
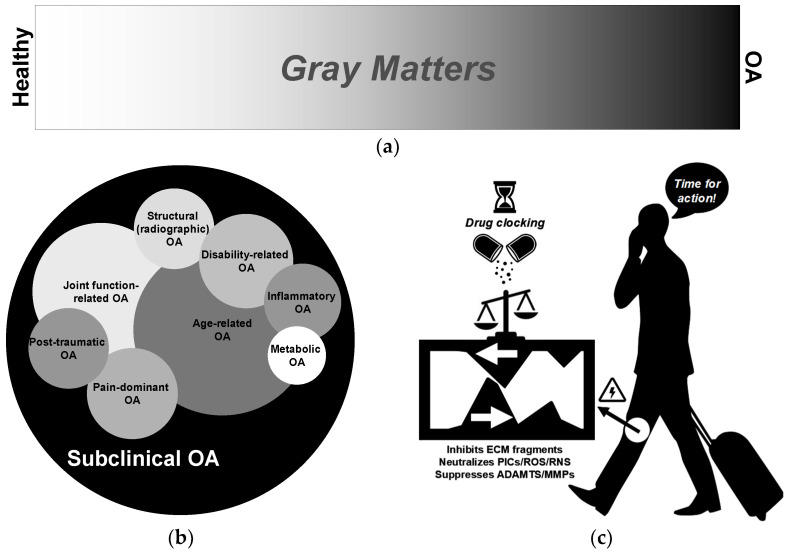
Characterizing the multidimensional spectrum and heterogeneity of OA phenotypes. (**a**) OA as a continuum beyond black-and-white paradigm. Rather than a dichotomy between health and disease, this panel depicts OA not as a binary state but as a continuous spectrum ranging from joint health (white) to advanced disease (black). The intermediate grey zone, often underappreciated, represents the clinical and biological heterogeneity of OA, capturing variable symptom burdens, structural changes, and rates of progression—highlighting the importance of nuanced diagnostic and therapeutic approaches. This gradient underscores the need for a more refined understanding of OA beyond traditional classifications. (**b**) OA pheno-endotypes and patient stratification for precision interventions. Illustrating the concept of phenotypic variation in OA, this panel emphasizes the multifactorial nature of the disease, encompassing mechanical, metabolic, inflammatory, and aging-related subtypes. Stratifying patients according to these distinct pheno-endotypes is crucial for optimizing treatment selection, trial design, and future implementation of personalized precision medicine in OA care. (**c**) Subclinical OA and the critical window for timely intervention. OA is often misunderstood to be a stable and easily manageable condition. However, it is characterized by significant variability in symptoms and progression [26]. Timing is a critical determinant of the efficacy of DMOAD therapy, particularly in progressive and heterogeneous diseases, such as OA. Focusing on the asymptomatic stage of OA, this panel emphasizes the concept of preclinical joint deterioration, where cartilage damage progresses silently in at-risk individuals (e.g., post-traumatic OA). This underscores the emerging opportunity to proactively intervene using chondroprotective agents and lifestyle modification, aiming to delay or prevent symptomatic disease onset and structural decline. By integrating early detection with systematic risk assessment, healthcare professionals, including community pharmacists, can drive informed decision-making for the timely initiation of DMOAD interventions, thereby potentially slowing disease progression and preserving long-term joint function. Abbreviations: ECM, extracellular matrix; PICs, pro-inflammatory cytokines; ROS, reactive oxygen species; RNS, reactive nitrogen species; ADAMTS, a disintegrin and metalloproteinase with thrombospondin motifs; MMPs, matrix metalloproteinases.

OA is traditionally viewed through a binary lens—either absent or present—yet this dichotomy oversimplifies its complex nature. Emerging evidence suggests that OA exists along a continuum, as schematically depicted in Figure 2a, where the “white” represents individuals without overt disease and “black” symbolizes those with advanced pathology, but the “grey” in between embodies a critical zone of heterogeneity. This gray spectrum reflects subtle variations in joint degeneration, symptom severity, and underlying pathophysiology, which are often masked by conventional diagnostic thresholds. Recognizing and characterizing these intermediate states are essential for developing targeted, personalized therapeutic strategies and for advancing our understanding of the multifactorial progression of OA. Recent advances in imaging and molecular diagnostics have begun to reveal the underlying pathomechanisms that contribute to this heterogeneity [139,140,168,169,170,171,172,173]. For example, magnetic resonance imaging (MRI) and biochemical markers now allow for a more nuanced assessment of joint cartilage integrity and inflammatory processes, facilitating the identification of intermediate disease states that were previously overlooked. Such technological advancements not only underscore the complexity of OA but also pave the way for stratified treatment approaches targeting specific disease phenotypes [168,172]. Understanding these gradations is critical, as it may help to elucidate why some patients experience rapid progression, while others maintain stable joint function despite similar radiographic findings. Ultimately, embracing this continuum model challenges traditional simplistic binary classifications and highlights the need for precision medicine in the management of OA, which remains a highly prevalent, disabling, and economically burdensome disease [4].

As shown in Figure 2b, OA is widely recognized as a heterogeneous disease with diverse clinical phenotypes and underlying pathological mechanisms [49,50,173]. While traditional views on OA have focused on symptomatic, advanced stages characterized by pain and functional impairment, a growing body of research highlights the presence of subclinical, asymptomatic OA. In these early stages, structural changes such as cartilage thinning, osteophyte formation, and subtle joint space narrowing occur without overt symptoms, often detectable only through advanced imaging techniques [174,175]. Examining subclinical OA from the perspective of aneural cartilage degradation is particularly evident, as cartilage deterioration is driven by a complex interplay of mechanical stress, biochemical mediators, and inflammatory processes (Figure 2c). ECM fragments, reactive oxygen/nitrogen species, pro-inflammatory cytokines—including TNF-α and IL-1β—and catabolic enzymes such as ADAMTS and MMPs [49,50,52,53,176] synergistically contribute to the early degradation of matrix components, thereby driving the progressive deterioration of cartilage. This asymptomatic phase challenges conventional diagnostic paradigms yet offers a critical window for ultra-early intervention. Consequently, it is essential to emphasize cartilage preservation through chondroprotective strategies that counteract these catabolic factors before irreversible damage occurs (e.g., collagen-fiber network), especially considering that effective cartilage regeneration requires multidimensional regulation of the biomechanical, inflammatory, and metabolic microenvironment within the joint—in both spatial and temporal dimensions—to create conditions favorable for repair [95,113]. Characterizing these early, pre-symptomatic stages and subclinical features of OA is critical for developing proactive, targeted interventions that can modify the disease course and prevent the onset of extensive irreversible joint damage and long-term disability.

Remarkably, after decades of intensive research, DMOADs continue to evade clinical validation, an outcome driven in part by the pervasive enrollment of patients with end-stage joint damage that is inherently refractory to reversal [74]. In contrast, emerging data indicate that the early subclinical phase of OA—marked by molecular and cellular alterations preceding radiographic or symptomatic changes—represents a critical window for intervention. A systematic review of two decades of preclinical knee OA models found that early prophylactic administration of glucosamine and chondroitin sulfate, particularly when incorporated into integrated multimodal approaches, enhanced cartilage integrity and favorably modulated key biochemical markers in a substantial proportion of studies, underscoring the promise of early chondroprotective intervention [41]. Supporting this, Drummen et al. recently demonstrated that supervised exercise and education programs in early knee OA significantly improve pain, function, and quality of life, highlighting the potential of non-pharmacological strategies to slow disease progression [177]. Recent studies have emphasized integrated multimodal regimens that combine chondroprotective agents (e.g., glucosamine and chondroitin sulfate) with personalized lifestyle changes and physical therapy, underscoring the need for rigorous evaluation to assess their potential synergistic efficacy in OA management [94,95]. When administered before significant cartilage loss, these agents may confer maximal benefit; however, most clinical trials enrolled patients with established OA, thereby constraining the observable impact on disease modification [41]. Redirecting clinical research toward the subclinical and early symptomatic stages could enable the development of interventions capable of altering the disease trajectory, delaying or even preventing the onset of debilitating joint damage. Such a paradigm shift would not only reduce the future burden of symptomatic OA but also mitigate its significant public health and economic costs [4]. To achieve this, rigorous investigation of chondroprotective agents in precisely phenotyped, early-stage cohorts is urgently needed, along with the development of advanced molecular and imaging-sensitive biomarkers for real-time monitoring for timely diagnosis and pharmacotherapeutic impact. By integrating mechanistic stratification, chronotherapeutic dosing, and interprofessional care pathways at the earliest stages of OA, it may be possible to shift the treatment paradigm from symptomatic palliation to true prevention of cartilage degradation and joint failure. Therefore, advancing our understanding and management of early-stage OA promises to transform care by preserving joint integrity, maintaining long-term function, reducing long-term healthcare costs, and markedly improving population-level outcomes.

Building on earlier points, pharmacists are strategically positioned to transform OA management through seamless integration of pharmacological and non-pharmacological strategies within a comprehensive, well-coordinated care framework. Their role extends beyond traditional dispensing functions to include comprehensive medication counseling, patient education, and the promotion of proactive care [14,20]. For example, pharmacists can identify asymptomatic individuals with a history of joint injury, particularly those at risk of developing subclinical cartilage damage and subsequent post-traumatic osteoarthritis (PTOA), and recommend early intervention with DMOADs [55]. By integrating such tailored pharmacotherapeutic approaches with lifestyle modifications and supportive non-pharmacological interventions, pharmacists may empower patients to play an active role in managing their condition, ultimately optimizing clinical outcomes and potentially altering the disease trajectory. However, for this potential to be fully realized, pharmacist communities must first be empowered—through advanced education, inclusion in multidisciplinary care models, and clear evidence-based protocols for DMOAD use. Enhancing the professional capacity of pharmacists and pharmacy technicians addresses current gaps in knowledge and practice, thereby improving the safe, timely, and effective delivery of novel therapeutic approaches. This supports the discussion in the subsequent sections.

## 8. Empowering Pharmacists in OA Care: Enhancing Patient Outcomes Through Education, Safety, and the What, How, and When of Strategic DMOAD Use

**Figure 3:**
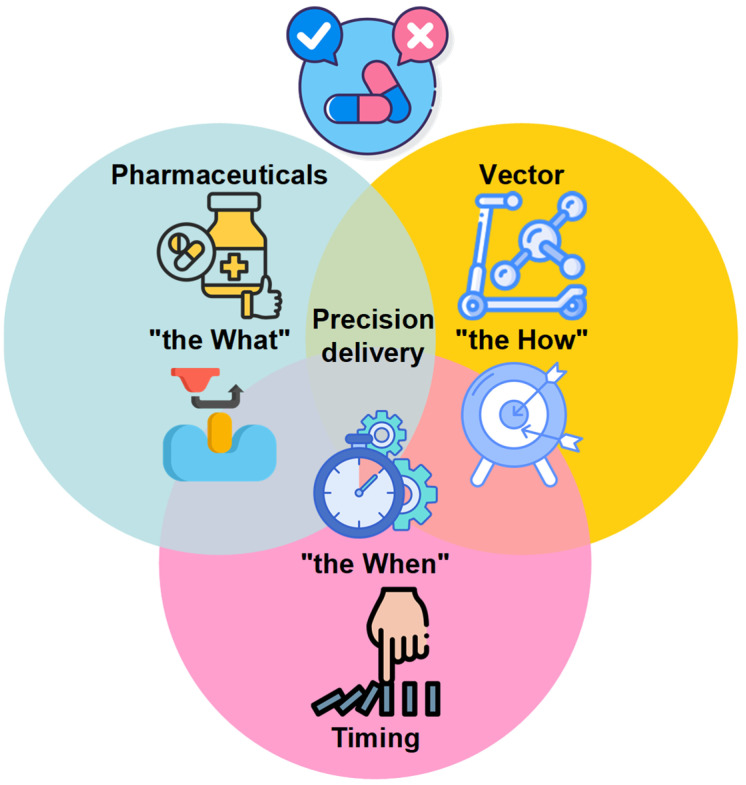
A conceptual Venn diagram illustrating an integrated DMOAD delivery framework for OA through three interdependent dimensions: “the What”—the chondroprotective agent (e.g., small-molecule inhibitors of cartilage degradation); “the How”—exploiting joint kinematics to drive shear-mediated diffusive transport from the synovial space into the cartilage interstitium; and “the When”—aligning systemic dosing schedules with both peak mechanical stress and circadian-regulated chondrocyte sensitivity to synchronize drug bioavailability with the optimal window for tissue uptake. Their intersection—Precision DMOAD Delivery—represents the convergence of payload, motion-enhanced transport, and rhythm-synchronized administration to maximize cartilage penetration, retention, and disease-modifying efficacy. This proof-of-concept tripartite model further embraces a holistic pharmacotherapy approach—combining evidence-based DMOAD regimens with complementary modalities and patient-driven lifestyle interventions—to maximize therapeutic efficiency, establish a more efficient and cost-effective healthcare delivery system, and clarify the ongoing debate over chondroprotective efficacy.

According to recent market analyses, millions of individuals worldwide incorporate glucosamine and chondroitin sulfate into their long-term OA management strategies [97,98,99,100]. As growing evidence supports the capacity of these chondroprotective agents to maintain cartilage integrity, pharmacists should ideally ensure that DMOADs achieve their full therapeutic potential. Drawing on their accessibility, pharmacological expertise, and patient-centered communication skills, community pharmacists can adopt a proactive role in OA care that integrates advanced disease-modifying approaches with patient education and risk assessment. As illustrated in Figure 3, this section introduces a comprehensive pharmacological framework for the strategic use of anti-OA drugs, organized around three critical dimensions: (i) defining therapeutic targets and chondroprotection criteria (“the What”); (ii) optimizing intra-cartilage drug delivery by leveraging joint biomechanics (“the How”); and (iii) establishing evidence-based timing for intervention (“the When”). Such a framework not only guides informed evidence-based decision-making but also empowers pharmacists to enhance patient outcomes through personalized education, rigorous safety surveillance, and the temporal alignment of therapeutic regimens with the biomechanical and circadian rhythms governing OA pathophysiology. By clarifying what constitutes effective DMOAD use, elucidating how to overcome inherent delivery challenges, determining the optimal timing for intervention, and emphasizing chondroprotective strategies that reduce reliance on NSAIDs and their associated risks, pharmacists can transform OA management from reactive symptom relief to a proactive, disease-modifying paradigm.

### 8.1. The What: Defining DMOADs and Their Role in OA Management

In OA, pharmacological chondroprotection encompasses the use of agents commonly classified as DMOADs (or SYSADOAs), which aim to preserve articular cartilage and slow disease progression rather than merely providing rapid symptomatic relief. These agents—primarily glucosamine and chondroitin sulfates—confer chondroprotective effects through multiple mechanisms, including inhibition of pro-inflammatory cytokines such as interleukin-1, restoration of extracellular matrix (ECM) components, enhancement of synovial fluid viscosity, and modulation of inflammatory signaling pathways by emerging phytonutraceutical compounds currently under investigation [30,37,38,43,44,79]. By maintaining the metabolic balance of the cartilage and the biomechanical integrity of the synovial joint, chondroprotective agents aim not only to reduce pain and enhance joint function but also to facilitate long-term structural remodeling, thereby delaying—or even preventing—the need for surgical interventions such as joint replacement.

As mentioned earlier, glucosamine sulfate and chondroitin sulfate are two of the most widely used chondroprotective compounds; they serve as building blocks for polysulfated glycosaminoglycans in cartilage and synovial fluid and exhibit anti-inflammatory actions that may slow osteoarthritic progression [29,30,178,179,180]. Glucosamine and chondroitin sulfate are endogenous matrix components essential for cartilage integrity. Glucosamine—an amino sugar derived from crustacean-shell chitin hydrolysis or microbial fermentation—is commonly administered as a sulfate salt, with its patented co-precipitated crystalline form achieving peak plasma and synovial concentrations of approximately 10 µM and a half-life of approximately 15 h at a daily dose of 1500 mg [181,182,183,184]. Chondroitin sulfate is a linear glycosaminoglycan of alternating N-acetylgalactosamine and glucuronic acid residues variably sulfated at the C4 or C6 positions; it is extracted from animal cartilages (bovine trachea, porcine ear/nose, shark and chicken keel) and purified via enzymatic digestion, fractionation, and precipitation to yield pharmaceutical-grade fractions (>90% purity, 10–40 kDa) [185,186,187,188]. Clinical guidelines typically recommend daily dosages of commercially available glucosamine sulfate (1500 mg) and chondroitin sulfate (800–1200 mg). In Europe, co-precipitated crystalline glucosamine sulfate and high-purity chondroitin sulfate have been approved as prescription drugs based on randomized trials, whereas in the United States, they are marketed primarily as dietary supplements without equivalent pre-marketing clinical requirements [28,36,69,121,122,124,125,185,189,190]. As nutraceuticals, they are available as OTC products; however, prescription-grade formulations may offer a more standardized and potent therapeutic option. The choice of salt form—sulfate versus hydrochloride for glucosamine sulfate and sodium versus calcium for chondroitin sulfate—critically influences solubility, stability, and bioavailability, and should be carefully considered in both clinical trial design and therapeutic use [101,121,122,124,129,130,133,183,185,191,192,193,194,195,196,197].

Importantly, both chondroitin sulfate and glucosamine sulfate have shown a good safety profile and are generally well tolerated with minor gastrointestinal reactions [34,36,43,135,182,198]. In vitro studies using human liver microsomes and recombinant cytochrome P450 (CYP) enzymes demonstrated that neither glucosamine sulfate nor chondroitin sulfate is appreciably metabolized by or inhibits CYP3A4 at concentrations up to several millimolar, indicating a minimal risk of cytochrome-mediated drug interactions [135,198]. Long-term safety appears acceptable for up to several years of use [27,32,34,43,45,128]. Raynauld et al. [32] robustly demonstrated that prolonged (≥2 years) glucosamine and chondroitin sulfate significantly mitigated cartilage volume loss in the lateral compartment of knees with baseline meniscal extrusion over six years in the Osteoarthritis Initiative (OAI) cohort, thereby underscoring the potential of these drugs as structure-modifying agents in knee OA—a paradigm that warrants validation through prospective randomized controlled trials to delineate optimal treatment duration and mechanistic pathways. A meta-analysis further supported the structural efficacy of chondroitin sulfate, demonstrating a modest but statistically significant reduction in joint space width loss over two years, particularly in studies using high-quality radiographic methods [79]. Collectively, these data underscore the plausible role of glucosamine and chondroitin sulfate as structure-modifying agents in knee OA, a therapeutic paradigm that warrants further validation through robust prospective randomized controlled trials to delineate the optimal treatment duration, responder phenotypes, and underlying mechanistic pathways.

However, despite the generally recognized safety of long-established SYSADOAs, such as glucosamine and chondroitin sulfate, concerns persist regarding their use in elderly patients, those with multiple comorbidities, and individuals on polypharmacy [39,40,199,200,201,202]. Although standard-dose glucosamine sulfate has been shown not to significantly alter fasting glucose, glucose tolerance, or insulin resistance in non-diabetic OA patients over 90 days [201], there remains theoretical and some preliminary clinical concern that in patients with untreated or poorly controlled diabetes, glucosamine—and potentially chondroitin—may exacerbate hyperglycemia and insulin resistance via the hexosamine biosynthesis pathway, underscoring the need for careful glycemic monitoring in diabetic populations receiving SYSADOAs [199]. Furthermore, the expert panel conclusively regarded glucosamine and chondroitin sulfate as inadvisable for patients with any degree of hepatic or renal impairment, underscoring the particular uncertainty surrounding chondroitin use in liver disease and a clear consensus that even mild kidney dysfunction precludes oral SYSADOA administration [39,40]. Notably, recent Mendelian randomization analysis suggests a potential causal relationship between chondroitin and glucosamine intake and reduced estimated glomerular filtration rate, raising further concerns about long-term renal safety and reinforcing the need for cautious use in individuals at risk of kidney dysfunction [202]. Additionally, a previous study involving 151 patients with chronic liver disease found that 15.2% had used glucosamine and/or chondroitin sulfate [200], and among these, two patients experienced significant elevations in liver enzymes temporally associated with glucosamine intake, suggesting a potential risk of hepatotoxicity, particularly in individuals with preexisting liver conditions, underscoring the importance of careful monitoring and reconsideration of their use in this comorbid population [25].

Building on our earlier discussion, DMOADs such as glucosamine sulfate and chondroitin sulfate are designed to go beyond symptomatic relief by directly intervening in OA pathogenesis—blocking key catabolic enzymes (ADAMTS, MMPs), modulating inflammatory cytokines, and counteracting oxidative stress to maintain cartilage integrity [29,30,52,178,179,180]. Although these agents have demonstrated benefits in some clinical trials, their effectiveness remains a subject of ongoing research and debate. To ensure the effective integration of DMOADs into routine clinical practice, explicit evidence-based criteria are required to define “how” and “when” these agents should be implemented in clinical practice. Establishing these criteria with an evidence-based rationale is essential for optimizing chondroprotective potential as part of an integrated OA treatment strategy.

### 8.2. The How: Implementing Strategic DMOAD Use in Clinical Practice

Regarding the challenge of intra-cartilage drug delivery, a question arises: are we truly mastering the biophysical barriers that separate DMOADs from their chondrocyte targets? Despite promising in vitro findings, the clinical efficacy of these agents remains unpredictable and inconclusive. Generally, effective pharmacological chondroprotection depends on overcoming the unique delivery challenges posed by avascular, aneural, and alymphatic hyaline cartilage. Once administered systemically, neutral small-molecule DMOADs must partition into the synovial fluid, cross a thin, unstirred boundary layer at the cartilage surface, and then navigate several millimeters of a dense, negatively charged proteoglycan–collagen network—where diffusion rates, as defined by Fick’s law, are constrained by molecular size, charge, partitioning characteristics, and local matrix properties. Most preclinical models, however, fail to replicate the non-agitated synovial microenvironment and its pharmacological “bottleneck”, leading to an overestimation of intra-cartilage drug uptake. Furthermore, OA-induced ECM remodeling and increased synovial viscosity exacerbate transport barriers in vivo, as degeneration of the superficial tangential zone (STZ) may further obstruct drug diffusion across the articular surface [203]. To close this critical knowledge gap, we must develop experimental systems that faithfully mimic joint biomechanics and synovial fluid dynamics, quantify the resistance imposed by the stagnant liquid film and lamina splendens in both healthy and diseased cartilage, and design DMOADs with optimized cartilage-targeting characteristics—thereby paving the way for truly rational, evidence-based chondroprotective therapy.

Given the challenge of achieving effective intra-cartilage drug delivery, it is crucial to recognize that the diffusion of chemical substances across biological membranes, including the dense proteoglycan–collagen network of the articular cartilage, is a fundamental yet severely constrained process. Although neutral small molecules are assumed to diffuse passively and permeate through the cartilage ECM, pioneering studies have demonstrated mass-transfer coefficients orders of magnitude lower than predicted [203,204,205], emphasizing that effective cartilage targeting demands drug candidates with rigorously optimized pharmacokinetic ADME(T) properties (absorption, distribution, metabolism, excretion (and toxicity)). Accordingly, orally administered DMOADs must sequentially traverse multiple biological barriers, including the intestinal mucosa, the synovial capillary endothelium, and the dense cartilage matrix, while retaining sufficient specificity, affinity, and bioavailability required for chondrocyte engagement [178,206]. However, beyond these well-characterized tissue barriers, heterogeneous physical obstacles, namely, unstirred synovial fluid layers that coat the cartilage surface, are likely to further impede solute transport by introducing an additional, potentially rate-limiting diffusional resistance, thereby adding uncertainty to in vivo drug penetration predictions. Despite their aqueous nature, these quiescent interfacial layers may significantly hinder drug transport by attenuating the concentration gradients and limiting solute replenishment at the cartilage boundary, thereby exacerbating the formidable challenges of intra-cartilage drug delivery. Even when drug candidates are engineered for optimal ECM penetration, this synovial fluid film imposes additional rate-limiting diffusional resistance at the cartilage surface, necessitating strategies to transiently disrupt this film and achieve therapeutically relevant intra-cartilage concentrations. Therefore, identifying, characterizing, and quantifying the combined effects of biological and physicochemical barriers may be indispensable for the advanced development of DMOADs with targeted tropism and permeability, as well as for designing rational delivery strategies capable of achieving consistent and clinically meaningful chondroprotection.

**Figure 4:**
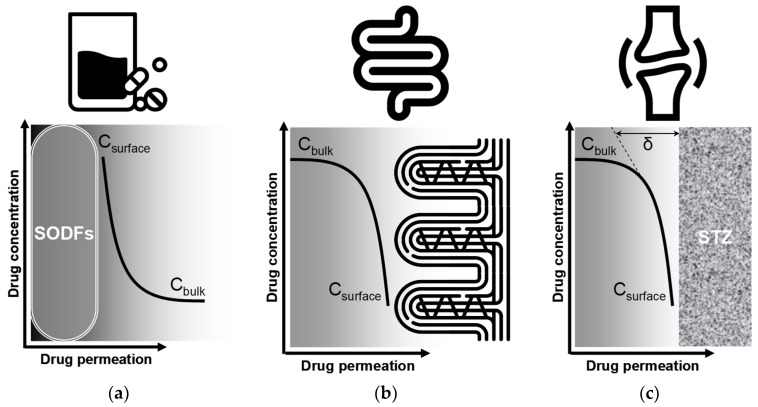
Schematic representation of serial stagnant-layer barriers to chondroprotective drug delivery: (**a**) a stagnant liquid film at the solid–liquid interface of an oral dosage form establishes the primary diffusional resistance controlling drug release; (**b**) an unstirred water layer immediately adjacent to the epithelial–vascular barrier imposes an additional diffusional resistance modulated by mucus, the glycocalyx, and peristaltic mixing, which constrains passive drug uptake into the circulation; (**c**) a low-mixing synovial fluid film overlaying avascular hyaline cartilage, introducing an upstream mass-transfer resistance prior to penetration of the negatively charged ECM. Here, δ represents the thickness of the unstirred layer, with arrows and lines indicating the diffusion pathways across this barrier. Together, these sequential stagnant films—from the formulation surface through the intestinal epithelium to the cartilage interface—define the concentration gradients and mass-transfer coefficients that govern dissolution, absorption, and intra-cartilage distribution. Moreover, each film creates a chemically distinct microdomain characterized by pH shifts, enzymatic degradation, and protein binding, which can transform, degrade, or sequester therapeutic molecules in ways that bulk assays fail to capture. These overlapping physical and chemical barriers produce spatial heterogeneity in drug penetration and significantly compromise solubility, stability, and therapeutic efficacy—challenges that are particularly critical for chondroprotective agents designed to establish high-affinity interactions and sustain effective bioactive concentrations at the chondrocyte interface. Adapted from [94,209,210,211,212,213,214,215,216,217,218,219,220,221,222,223]. Abbreviation: SODFs: Solid Oral Dosage Forms; STZ: superficial tangential zone.

The diffusion transport of chemical substances across biological membranes, such as the articular cartilage [207,208], is essential in all forms of life. From the aqueous dissolution of oral dosage forms, where a stagnant liquid film at the solid–liquid interface dictates the rate at which a drug enters solution, to the intestinal unstirred layer that governs solute uptake by the epithelium, stagnant boundary films impose a first line of diffusional resistance that shapes bioavailability (Figure 4). Similarly, in diarthrodial joints, a thin, low-mixing synovial fluid film covers the cartilage surface, adding a further diffusion barrier upstream of the dense, negatively charged ECM. These serial resistances—from the solubility-limiting film around an oral formulation, through the intestinal diffusion boundary layer, to the cartilage-surface fluid film—collectively determine the kinetics and depth of intra-tissue drug delivery from [94,209,210,211,212,213,214,215,216,217,218,219,220,221,222,223]. A mechanistic understanding of how stagnant layers alter concentration gradients and mass-transfer coefficients is therefore pivotal for rational formulation design, predictive in vitro testing, and the development of delivery strategies (including carrier engineering and physiologically timed joint motion) that overcome these composite barriers. Beyond their well-characterized function in physical diffusion resistance, unstirred boundary films also establish unique chemical microenvironments that can dramatically alter drug stability, speciation, and bioavailability. Within these stagnant films, steep pH gradients—arising from localized ion transport and limited buffer exchange—may shift the ionization state and solubility of pH-sensitive therapeutics [210,224,225]. Concurrently, the immobilized milieu concentrates endogenous enzymes (e.g., peptidases in the gut or proteolytic and oxidative species in synovial fluid) and ECM components, facilitating site-specific degradation or binding of therapeutic molecules prior to tissue permeation. Furthermore, synovial proteins enable non-covalent binding and sequestration, effectively reducing the fraction of free, diffusible pharmaceuticals available for cartilage penetration. In parallel, surface-associated and fluid-phase solutes within the unstirred layer may enzymatically or chemically modify bioactive compounds, such as glucosamine, vitamin D precursors, or small-molecule therapeutics, thereby altering their solubility, bioactivity, or molecular form prior to entry into deeper tissue zones. Such chemical interactions within the unstirred layer may therefore modulate the free drug concentration at the epithelial or cartilage surface, distort the apparent permeability or kinetic parameters, and introduce non-uniform exposure profiles that cannot be predicted by bulk assays alone. Obviously, these phenomena add further complexity and variability in drug performance, particularly for chondroprotective agents, whose efficacy is often debated. For these bioactive compounds, where efficacy hinges on achieving and sustaining therapeutic levels within the avascular cartilage matrix, these coupled physical and chemical phenomena in the diffusion boundary layer represent critical yet often underappreciated determinants of bioavailability and clinical outcome.

Building on the interplay of biological and physicochemical barriers, the synovial unstirred layer—or stagnant liquid film—at the cartilage surface represents a critical diffusion-limited zone that all prospective DMOADs must traverse. By definition, an unstirred layer is a thin region of fluid immediately adjacent to a solid interface, where convective motion is negligible and solute transport occurs solely by molecular diffusion, a phenomenon arising from the nonslip condition at boundaries [209,210]. In synovial joints, this layer comprises a viscoelastic, non-Newtonian fluid predominantly consisting of approximately 95% water, 2.5% high-molecular-weight hyaluronic acid, 1.5% proteins, and trace amounts of glucose, lipids, and electrolytes. Of particular significance are matricial fragments—such as COMP, FN-fs, Helix-II, or CTX-II—which are hypothesized to adhere to the lamina splendens and STZ of the cartilage ECM, potentially influencing cartilage biomechanics and molecular transport. First described by Maroudas et al. [204] in the late 1960s, stagnant films concentrate the solutes under minimal joint motion and thereby can dramatically slow down drug permeation. Recent investigations have quantified thickness, rheological behavior, and transient solute–film interactions, underscoring the necessity of incorporating this natural barrier into predictive models and delivery strategies for effective intra-cartilage pharmacotherapy [226]. These findings highlight the need to integrate accurate descriptions of interfacial transport barriers when designing effective strategies for intra-cartilage drug delivery.

Drawing upon the biophysical framework of intra-cartilage transport, the synovial unstirred layer can be described using classic film theory as a homogeneous diffusion barrier whose thickness (δ) directly constrains the flux (J) of chondroprotective agents. According to Fick’s first law, adapted for stagnant films:

[J = D (C_bulk_ − C_surface_)/δ] (1)

where D is the molecular diffusion coefficient, C_bulk_ is the drug concentration in well-mixed synovial fluid, and C_surface_ is the concentration immediately adjacent to the cartilage surface. In vivo, δ—and hence J—are dynamically regulated: joint movement or synovial mixing thins the unstirred layer, whereas increased fluid viscosity or lower diffusivity thickens it, with boundary-layer hydrodynamics predicting δ∝D1/3 under laminar conditions [210,213,227]. Because this stagnant film lies in series with the dense proteoglycan–collagen matrix, even modest increases in δ can dramatically decrease the concentration gradient driving trans-cartilage diffusion of a pharmacologic agent, thereby reducing net flux and effective permeability [204,226]. Such an overlooked rate-limiting step likely contributes to the inconsistent clinical translation of in vitro chondroprotective success—exemplified by glucosamine sulfate and chondroitin sulfate—into tangible patient benefits. Yet, despite extensive cartilage solute transport research [207,208], most experimental and computational models still overlook the unstirred synovial layer, underscoring the urgent need to integrate its diffusion-limiting characteristics into molecular transport equations and to tailor the DMOAD design that can overcome this critical barrier to effective, evidence-based chondroprotection.

**Figure 5:**
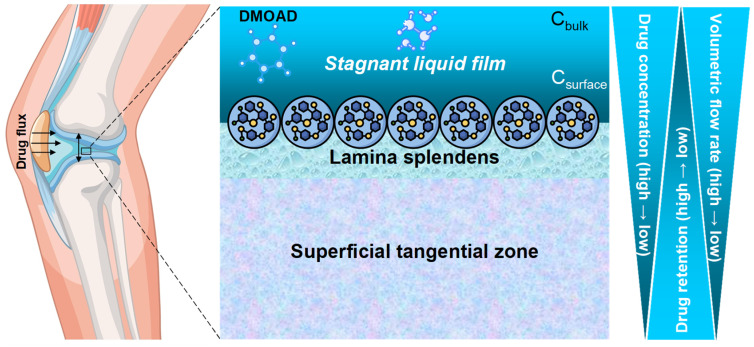
Cross-sectional schematic of the human knee articular surface, highlighting three successive diffusion barriers to intra-cartilage drug delivery: (i) a thin, nearly stagnant synovial fluid film (50 to >1,000 µm) that, under low-flow conditions, imposes the initial mass-transfer resistance and limits replenishment of low-molecular-weight therapeutics at the cartilage interface; (ii) the lamina splendens, a 5–10 µm acellular, amorphous collagenous coat with superlubricity (COF ~0.005) and ultra-low hydraulic permeability, which provides a secondary seal and boundary lubrication while further restricting solute entry; and (iii) the STZ (100–200 µm), composed of densely aligned type II/IX collagen fibrils, proteoglycans, and interstitial water, whose highly charged, tortuous ECM drastically reduces effective diffusivity, excludes anionic molecules through Donnan partitioning, and may sequester, unevenly distribute, or entirely block cationic drugs. These serial fluid film and matrix resistances produce steep concentration microgradients and marked spatial heterogeneity in drug distribution, underscoring the critical need for mechanical stimulation or advanced carrier design to overcome these barriers and ensure effective chondroprotective therapy.

Enhancing the intra-cartilage delivery of fast-cleared small-molecule DMOADs depends on our ability to modulate the unstirred synovial layer through articulation biomechanics. Joint motion (physical activity/exercise or physiotherapy) thins δ—shifting the delivery bottleneck to the drug residence time—whereas immobilization or inflammation (synovitis) thickens it, causing molecular diffusion to cease before therapeutic concentrations are achieved. To translate the promising results of in vitro studies of chondroprotective agents into consistent clinical outcomes, we need to establish evidence-based protocols that conceptualize the synovium as the “placenta” of avascular cartilage and intentionally leverage joint motion as a drug delivery strategy (Figure 5). Such protocols could include prescribing targeted joint-movement regimens; timely post-dosing to maximize synovial mixing [94,95]; co-formulating DMOADs with excipients that modulate barrier properties; engineering molecules with cartilage-affinity, enzyme-responsive triggers, or rheology-altering domains [221,228,229]; and deploying innovative techniques—like ultrasound or microbubble cavitation [229,230,231,232,233]—to transiently reduce δ. By incorporating unstirred-layer parameters into diffusion models, synovial fluid rheology into pharmacokinetics, and patient-specific biomechanics into clinical trial designs, we can bridge the bench-to-bedside gap, enhance SYSADOA/DMOAD efficacy in next-generation studies, and ultimately improve outcomes in individuals with OA.

In summary, we engineered DMOADs with potent chondroprotective mechanisms; therefore, why are we still “stagnant” at the cartilage boundary? In addition to their classical role as mere physical diffusion barriers, unstirred layers constitute localized microenvironments in which chemical transformations, such as pH-dependent solubility changes, enzymatic degradation, and drug–excipient or drug–protein interactions, can occur. These phenomena may exacerbate transport heterogeneity and introduce pharmacokinetic variability, which are likely to contribute, at least in part, to the inconsistent clinical efficacy of chondroprotective therapies. Shockingly, how can we claim truly rational pharmacological chondroprotection if we continue to ignore the stagnant liquid film between the active molecule and target? It is remarkable—and regrettably true—that after decades of cartilage transport research, we still behave as if unstirred layers do not exist. Until the diffusion-limiting blueprint is fully integrated into predictive models, DMOAD development, and clinical trial design—and cyclical joint motion is deliberately utilized to transiently thin this barrier—the pursuit of effective chondroprotection will likely remain impeded by a critical blind spot, warranting further investigation.

### 8.3. The When: Optimizing the Timing of DMOAD Intervention

Articular cartilage functions as a highly specialized biomechanical interface, where a precise equilibrium between chondrocyte metabolism and ECM turnover sustains joint load distribution and minimizes friction [47,48]. However, this equilibrium can be rapidly disrupted by subtle alterations in mechanical loading, inflammatory mediators, or solute transport, initiating a cascade of matrix degradation and cellular dysfunctions. Such perturbations underscore the vulnerability of cartilage homeostasis and highlight the need to elucidate the molecular and biophysical mechanisms governing its resilience. A deeper understanding of these processes is essential for the development of targeted interventions to prevent or reverse the progressive degeneration characteristics of OA.

Cartilage possesses an intrinsic circadian clock that regulates daily cycles of matrix turnover and metabolism. Chondrocyte clock genes (e.g., Bmal1, Per, Cry) drive oscillations in protease expression and ECM synthesis [234,235,236,237,238,239,240,241]. Proteomic studies have shown that cartilage degradation processes peak in the early morning hours, whereas anabolic activities (e.g., proteoglycan and collagen synthesis) predominate in the afternoon and overnight [237]. Likewise, biomarkers of cartilage turnover (COMP, hyaluronan, CTX-II, etc.) exhibit diurnal rhythms, with the highest levels measured in the morning. Mechanistically, the circadian clock in chondrocytes coordinates the energy metabolism and proteolysis. For example, proteasomal degradation peaks at dawn, whereas ATP production and matrix synthesis increase during the evening and night. Disruption of these biorhythms (e.g., by mechanical stress) contributes to OA pathogenesis [238], indicating that maintaining synchrony is critical for cartilage homeostasis. Indeed, the patterns of daily biomechanical stimuli on synovial joints also follow a reproducible rhythm. These mechanical surges entrain and influence the cartilage clock; cyclic loading and osmotic changes act as “zeitgebers”, resetting chondrocyte rhythms [238,239,240]. In vitro experiments have confirmed that rhythmic compressive strain can shift cartilage clock gene expression (for example, Clock, Bmal1, Per2) and even induce anabolic responses in concert with mechanical cues [234,235,236,237], thereby underscoring the potential of precisely timed mechanical interventions to optimize tissue repair.

Given this chronobiology, chondroprotective interventions can be synchronized to the window of the greatest cartilage vulnerability. Recently, an “early-morning chondroprotective phase” has been described, in which elevated matrix-degrading activity makes timely drug delivery particularly beneficial [95]. Accordingly, this chronopharmacological model advocates the administration of oral agents (e.g., glucosamine and chondroitin) with breakfast, ensuring that systemic drug exposure peaks coincide with the morning catabolic increase, potentially maximizing therapeutic benefit. This “same-phase” strategy may directly counteract the circadian degradation wave: by aligning drug bioavailability with early-morning upregulation of MMPs, ADAMTS, and inflammatory cytokines, therapy can attenuate matrix breakdown when it is most active [95]. Conversely, dosing in the evening (when catabolic activity is low) creates a mismatch between drug peak and cartilage vulnerability, likely reducing efficacy. In practical terms, this means recommending morning dosing of slow-acting chondroprotectives so that their absorption and tissue uptake overlap with the hours of greatest mechanical joint loading and cartilage catabolism [95]. This concept mirrors photoprotection: just as sunscreen must be applied before peak UV exposure to ensure maximal skin defense [242,243], chondroprotective agents should be administered orally to establish therapeutic concentrations in the synovial joint prior to or concurrently with—but not after—periods of maximal mechanical stress, thereby ensuring optimal joint protection.

**Figure 6:**
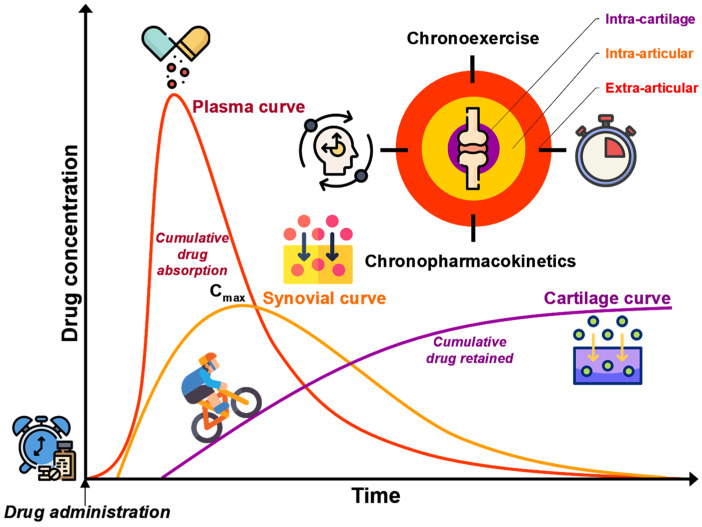
Chronopharmacology in motion: drug concentration–time curves for plasma (red), synovial fluid (yellow), and cartilage (purple). This schematic illustrates the sequential pharmacokinetics of an orally delivered chondroprotective agent, including rapid systemic absorption and peak plasma levels, transient equilibration within the synovial compartment, and cartilage penetration. Systemic delivery is constrained by rapid synovial clearance and restricted diffusion into—and suboptimal absorption by—the cartilage matrix, thereby compromising therapeutic intra-cartilage concentrations. Strategically, timing joint movement to coincide with peak synovial concentrations harnesses convective forces in the synovial fluid, prolongs joint residence time, and enhances advective penetration into the cartilage. This integrative approach, which synchronizes pharmacokinetic maxima with joint motion, constitutes a mechanistic framework for optimizing DMOAD delivery and efficacy in OA.

Synovial liquid turnover—on the order of one hour—together with lymphatic clearance poses a formidable obstacle to sustaining effective concentrations of low-molecular-weight (LMW) chondroprotectives in the joint space. Orally administered active molecules, such as pharmaceutical-grade glucosamine sulfate or LMW-chondroitin sulfate, are typically cleared from the synovial fluid within hours [244,245], severely restricting cartilage exposure. Beyond rapid clearance (Figure 6), the dense collagen–proteoglycan network of cartilage and the unstirred synovial film at its surface further limit passive diffusion into deeper zones [204,210,213,226]. Importantly, the dynamic joint motion can overcome these diffusion barriers. The hydrodynamic pressures generated by sliding joint articulation can enhance synovial fluid mixing, disrupt stagnant boundary films, and promote advective solute transport into the cartilage matrix. In vitro fluorescent tracking with confocal microscopy has shown that sliding at 60 mm/s enhances solute uptake by several orders of magnitude compared with static diffusion or cyclic compression alone [246,247]. Specifically, Graham et al. [246] demonstrated rapid penetration into buried contact regions under sliding, an effect not observed at slower speeds, while Culliton et al. [247] quantified a 2.1-fold and 4.4-fold increase in uptake after 30 min and 2 h of sliding, respectively, versus axial loading. Translational pharmacokinetic models further highlight how synovial fluid dynamics govern drug fate in diarthrodial joints [245], underscoring the need to integrate exercise-timed interventions with pharmacotherapy. These insights have significant implications for maintaining cartilage health and for developing therapeutic strategies for OA and other degenerative joint conditions. Clinically, initiating low-impact exercises, such as pedaling within three hours of DMOAD ingestion, may align peak plasma–synovial concentrations with maximal fluid mixing, thereby optimizing cartilage delivery [95]. Furthermore, timing of pharmacological treatment to coincide with routine activity patterns can leverage joint mechanics for sustained drug retention and distribution [94]. Thus, in light of the dual role of synovial fluid—not only nourishing cartilage but also mediating drug transport—coordinating exercise-timed regimens with the articular pharmacokinetic profile of chondroprotective agents provides a mechanistically rational strategy to overcome rapid clearance and diffusion limitations, ultimately optimizing therapeutic efficacy in OA management.

As previously discussed, effective agitation of synovial fluid may be critical for the intra-cartilage delivery of orally administered pharmacologic agents. While active pedaling can serve as a practical method to enhance synovial fluid mixing, its feasibility is often limited among patients with knee or hip OA due to significant pain, functional impairments, and kinesiophobia—a fear of movement driven by pain avoidance and misconceptions about joint vulnerability. These individuals are frequently older, predominantly female, and may exhibit muscle weakness, frailty, and multiple comorbidities that further restrict voluntary exercise [25,26]. In addition, chronic sedentary behavior, often associated with overweight or obesity, induces aberrant mechanical loading that diminishes cartilage matrix permeability, as demonstrated by the inverse relationship between compressive loading and solute diffusion in joint cartilage [205,248]. In this context, motor-driven passive cycling provides a clinically viable alternative that enables controlled synovial mixing and potentially enhances drug distribution without mechanical stress, thereby supporting drug delivery under conditions unlikely to provoke inflammation. Putatively, this passive cycling modality may serve as a reliable orthopedic strategy for cartilage-targeted drug delivery, minimizing joint strain and facilitating the penetration and distribution of orally administered chondroprotective agents into articular cartilage. Importantly, passive motion may be particularly beneficial for patients with impaired motor control or exercise intolerance, supporting targeted drug delivery under low-risk conditions. By tailoring parameters such as pedaling speed, range of motion, and session duration to individual tolerance, passive cycling can maximize synovial mixing while minimizing patient discomfort. Once pain and inflammation are adequately managed and joint tolerance improves, the ultimate goal is to restore physiological cyclic loading (e.g., active cycling exercise, walking, or stair climbing) to support long-term cartilage homeostasis and functional recovery. Further research is required to validate these passive cycling protocols and define the optimal biomechanical parameters for targeted intra-cartilage drug delivery.

In conclusion, aligning oral chondroprotective therapy with the intrinsic circadian rhythm of cartilage and patient activity cycle may enhance therapeutic efficacy. The collective evidence supports preferential morning dosing: by delivering drugs at the outset of daily activity, we “meet the clock” of cartilage catabolism and neutralize its effects in real-time [95]. This chronotherapy approach is bolstered by multiple lines of recent research on cartilage chronobiology and joint mechanics [237,238,246]. Future clinical studies—guided by the rigorous design principles established in the ATLAS trial [44], which assessed a synergistic combination of Boswellia serrata extract, pine bark extract, curcumin, piperine, and methylsulfonylmethane—should comprehensively evaluate whether chronotherapeutic dosing of agents such as glucosamine, chondroitin, novel DMOADs, small-molecule inhibitors, antioxidants, nutraceuticals, phytonutrients, and Mediterranean diet-derived phytochemicals (including flavonoids, polyphenols, and carotenoids) provides superior symptomatic and structural outcomes compared to conventional fixed dosing schedules. Such studies will further refine these timing recommendations and advance the precision chronomedicine paradigm in OA management.

## 9. Challenges, Applications, and Future Perspectives

Pharmacies play a pivotal role as accessible healthcare touchpoints for individuals managing early symptoms of OA, often serving as the first contact for those seeking pain relief through OTC analgesics or anti-inflammatory medications [9,10,14,20]. While community pharmacies are well-positioned to act as gatekeepers or early-warning systems, their role is most effective when integrated into a collaborative healthcare framework [249]. Pharmacists can enhance OA management by offering basic screening for joint pain, educating patients about OA risk factors and progression, and guiding those with persistent or worsening symptoms to seek formal medical evaluation. Furthermore, by monitoring medication usage patterns, they can identify potential overreliance on painkillers or anti-inflammatory agents and relay these insights to healthcare providers for further intervention. However, the notion that pharmacies operate as standalone pharmacovigilance centers may overstate their current diagnostic and longitudinal tracking capabilities. Empowering pharmacists with additional training and fostering stronger connections with healthcare providers could significantly enhance early detection, education, and management of OA within the community. These preliminary considerations highlight both the challenges and promising opportunities for enhancing the role of pharmacies in OA care, underscoring that unlocking this potential will require coordinated strategies that integrate clinical expertise, patient education, and health system collaboration to deliver comprehensive, person-centered care.

### 9.1. Implications for Community Pharmacy Practice

Community pharmacies occupy a unique position at the intersection of medication access, patient education, and chronic disease management, making them ideally suited for delivering proactive chondroprotective care in OA [8,9,10,11,12,13,14,15,16,17,18,19,20,21,24,250]. By integrating brief screening for OA risk factors, such as age, body mass index, prior joint injury, joint pain duration, and functional limitations, into routine OTC consultations or prescription pick-ups, pharmacists can identify individuals who may benefit from DMOADs and initiate a focused dialogue on both symptomatic relief and long-term cartilage preservation [20,190]. Employing validated, patient-friendly tools such as a simplified pain–function questionnaire enhances the accuracy of these encounters without disrupting workflow, while stratifying patients by OA phenotype and endotype enables early recognition of subclinical disease states that warrant pre-symptomatic intervention. By defining these parameters, these professionals can provide tailored recommendations—particularly regarding glucosamine and chondroitin sulfates—to optimize joint health from the earliest stages of OA and improve patient outcomes.

Pharmacists are highly accessible frontline providers, often representing the first point of contact within the healthcare system. Following physicians and nurses, pharmacists constitute the third largest professional group globally [12]. While adults typically consult their primary care physician four to seven times annually, they may visit community pharmacies as many as 35 times per year, resulting in approximately five-fold more interactions with pharmacists than with family doctors [17,21]. Given that nearly 60% of individuals with clinically diagnosable OA may not actively seek formal medical attention [20], frequent, low-threshold encounters with pharmacists offer a critical opportunity for early identification and outreach. Despite this potential, there is currently a lack of data quantifying the frequency and nature of OA-specific inquiries in community pharmacies, underscoring the need for targeted research to inform service planning and delivery in this area. Such regular patient engagement enables pharmacists to contribute meaningfully to the management of chronic diseases and public health initiatives [13,16]. By leveraging these regular touchpoints, they can reinforce patient education on chondroprotective strategies, monitor adherence to DMOAD regimens, and identify potential safety issues, thereby embedding OA care into the daily routines of affected individuals.

Monitoring and follow-up are critical for ensuring both the efficacy and safety of long-term DMOAD therapy. Community pharmacists can implement structured touchpoints at 1, 3, and 6 months post-initiation to systematically assess patient-reported outcomes (e.g., pain via VAS, function via simplified WOMAC), adherence patterns using pill counts or digital reminders, and the emergence of gastrointestinal, metabolic, or other adverse events, leveraging simple adherence-tracking tools or digital reminders when available. If patients experience red-flag symptoms, such as deteriorating glycemic control in diabetes or elevation in hepatic transaminases, pharmacists should recommend prompt laboratory evaluation or specialist referral, particularly in individuals with existing renal or hepatic compromise where DMOAD use requires rigorous pharmacovigilance [39,40]. By embedding these proactive follow-up protocols into routine practice, these trusted providers not only safeguard patient safety, but also reinforce adherence and maximize the structural benefits of chondroprotective therapy.

High patient health literacy is a cornerstone of successful OA management, enabling individuals to engage actively with chondroprotective strategies [162,163,164,165,166,167]. Within the community pharmacy context, this involves translating the pharmacological intricacies of DMOADs into straightforward patient-centered guidance that supports informed decision-making. Pharmacists should first clarify “what” these agents are—therapeutics designed to maintain cartilage homeostasis rather merely attenuate pain—and emphasize their well-characterized safety profile, including negligible cytochrome P450 interactions that reduce the risk of drug–drug interactions [135,198]. Importantly, they should highlight the critical distinction between commonly available non-standardized supplements and pharmaceutical-grade prescription formulations, emphasizing the rigor of quality assurance, consistent dosing, and robust clinical evidence that supports therapeutic reliability. Next, they can describe “how” these agents function, underscoring that encouraging mild joint movement after dosing enhances synovial fluid mixing and facilitates drug diffusion through the avascular cartilage matrix to reach chondrocytes. Finally, advising patients on “when” to administer chondroprotective agents within chronotherapeutic guidance can further optimize their efficacy; for instance, recommending morning administration of glucosamine sulfate ensures that peak plasma levels coincide with early morning upregulation of catabolic enzymes, thereby harnessing circadian rhythms to mitigate cartilage matrix degradation [95]. Framing these principles with familiar analogies—such as comparing scheduled DMOAD intake to applying sunscreen before peak sun exposure—pharmacists can make complex dosing schedules more relatable, thereby enhancing patient understanding and health literacy, and foster the consistent adherence needed to achieve optimal therapeutic outcomes.

Despite their wide availability as OTC nutraceuticals, glucosamine and chondroitin products vary considerably in purity, salt form, and bioavailability [101,121,122,124,129,130,133,183,185,191,192,193,194,195,196,197]. Pharmacists should guide patients to prescription-grade formulations, such as crystalline glucosamine sulfate and pharmaceutical-grade chondroitin sulfate, which are manufactured under stringent quality control protocols and have demonstrated consistent pharmacokinetic properties and clinical efficacy in randomized controlled trials [28,41,121]. By addressing cost-related adherence barriers through tailored discussions of insurance coverage plans, generic substitution options, and co-pay assistance resources, pharmacists can significantly enhance patient persistence with chronic DMOAD regimens and optimize long-term therapeutic outcomes. Furthermore, adapting educational materials to the cultural background and health literacy level of each patient enhances comprehension and engagement, reduces the likelihood of self-medication with non-standardized supplements or suboptimal preparations, and promotes the safe, evidence-based use of slow-acting chondroprotective agents [36].

Effective pharmacotherapy remains a cornerstone of conservative OA management, with treatment strategies broadly divided into symptom-modifying agents (e.g., NSAIDs, acetaminophen) that provide rapid analgesia and disease-modifying drugs, such as glucosamine and chondroitin sulfate, which aim to preserve cartilage integrity and slow structural progression [74]. However, as previously discussed, the efficacy of orally administered DMOADs may be limited by stagnant synovial fluid films that impede drug diffusion into avascular cartilage, raising critical questions about optimal dosing schedules—morning, noon, evening, or bedtime—and regimen selection for patients with predominantly nocturnal or morning pain. Chronotherapeutic considerations suggest that NSAIDs and acetaminophen, which target well-perfused synovial and subchondral tissues, may achieve maximal benefit when administered in the late evening to alleviate nighttime symptoms and improve sleep quality [77,251], whereas DMOADs directed at cartilage may be most effective when taken in the morning, leveraging daytime joint movement to enhance synovial mixing and counteract the catabolic circadian peak of chondrocytes [234,235]. To reduce patient uncertainty, support adherence, and optimize outcomes, drug information leaflets and package inserts should include explicit dosing-time recommendations along with clear explanations of the mechanistic rationale. Recognizing the synergistic interplay between chronopharmacological mechanisms and molecular transport within articular cartilage, it is imperative that future product labeling incorporates succinct, evidence-based recommendations on adjunct physical activity and physiotherapeutic interventions—specifically, prescribing joint mobilization exercises within the initial three hours post-administration, alongside targeted load management strategies. Such guidance would enable pharmacists to provide standardized, guideline-consistent counseling at the point of sale, thereby supporting patients in adopting integrated medication–lifestyle interventions to achieve optimal therapeutic outcomes.

To sustain and broaden their contribution in OA care, community pharmacists should actively engage in targeted continuing education covering DMOAD pharmacology, chronotherapeutic dosing, adverse event surveillance, interprofessional collaboration, and patient-centered communication. Accredited programs offered by organizations such as the International Pharmaceutical Federation (FIP), national pharmacy associations, and dedicated OA consortia deliver the latest clinical practice recommendations and practical workshops. Complementing these efforts, pharmacies can disseminate educational patient-facing materials developed by reputable international and regional authorities to strengthen patient engagement and enhance the uptake of evidence-based interventions [28,35,42,91,93,122,125,190,252]. These include clinical guideline organizations (e.g., Osteoarthritis Research Society International [OARSI], American College of Rheumatology [ACR], European Alliance of Associations for Rheumatology [EULAR], and the European Society for Clinical and Economic Aspects of Osteoporosis, Osteoarthritis, and Musculoskeletal Diseases [ESCEO]), regional rheumatology networks (e.g., Asia-Pacific League of Associations for Rheumatology [APLAR], Pan American League of Associations for Rheumatology [PANLAR], and the British Society for Rheumatology [BSR]), and leading patient advocacy groups (e.g., Osteoarthritis Action Alliance [OAAA], Arthritis Foundation, Arthritis Australia, Osteoarthritis Foundation International [OAFI], Canadian Arthritis Patient Alliance [CAPA], and Versus Arthritis). By integrating these educational resources—ranging from concise brochures and data-driven infographics to interactive digital platforms such as mobile apps and web-based decision-support tools—into routine counseling practices, pharmacists can enhance patient understanding, elevate health literacy, reinforce adherence to chondroprotective therapies, and promote informed, self-directed OA management that aligns with evidence-based best practices.

Building on the need for comprehensive professional development and patient resources, a critical gap remains: despite extensive guidance from leading rheumatology and musculoskeletal organizations, no specific evidence-based guideline directs the rational use of DMOAD agents in OA care. Educating patients on optimal drug application—including chronotherapeutic timing, expected onset of benefit, and safety monitoring—is essential to maximize efficacy and minimize misconceptions [22,142,144,147,150,157,158]. As mentioned earlier, community pharmacists are uniquely positioned to bridge this gap because of their accessibility and pharmacological expertise. However, current educational frameworks rarely include specialized training in chronotherapy, regimen individualization, or collaborative decision-making. Without these competencies, these frontline professionals cannot consistently deliver the nuanced patient-centered guidance required for effective OA management. Addressing these educational deficiencies is essential for pharmacists to optimize the use of chondroprotective agents [94,95]. Accordingly, developing and integrating practical, DMOAD-specific guidelines into both pre-licensure curricula and continuing education programs will empower pharmacists to deliver safe, effective, and truly holistic patient-centered chondroprotective care.

Looking ahead, community pharmacists and pharmacy assistants are ideally positioned to lead practice-based outcomes research on DMOAD effectiveness by leveraging digital adherence platforms, telepharmacy consultations, and integration with electronic health records to capture robust real-world data. By systematically documenting outcomes, monitoring safety and cost-effectiveness, and analyzing these findings, pharmacies can iteratively refine patient education, tailor chronotherapeutic strategies, and develop practical guidelines. In doing so, we will establish a precision chronomedicine paradigm for OA, ultimately improving the long-term joint health and quality of life of millions of patients affected by this chronic disease.

### 9.2. CHONDROMOVING—A Visual, Lifestyle-Integrated Brochure Prototype for Enhancing OA Medication Safety and Efficacy

**Figure 7:**
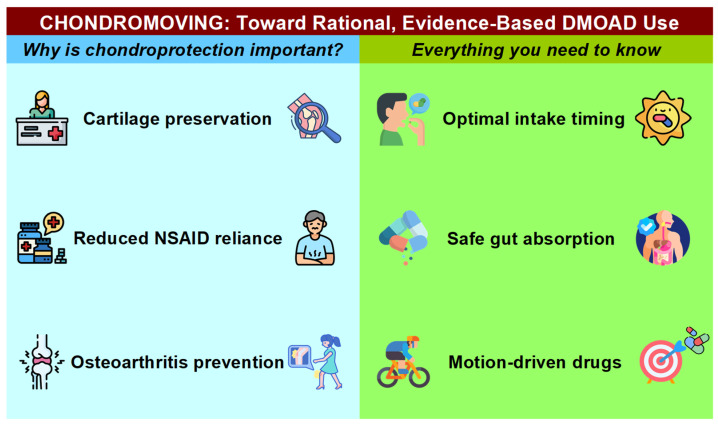
Prototype CHONDROMOVING brochure for precision-guided pharmacological chondroprotection. This two-panel, infographic-driven layout integrates medication and lifestyle guidance. The left panel (“What”) depicts the joint-specific targets of prescription-grade glucosamine sulfate and high-purity chondroitin sulfate alongside a gastrointestinal safety icon and a brief monitoring advisory. The right panel (“How/When”) uses clock and exercise symbols to depict post-dose joint mobilization and morning dosing synchronized with circadian enzyme peaks—strategies that enhance synovial fluid mixing, improve cartilage penetration, and prolong intra-cartilage residence time. A supplemental reverse side can feature a symptom-tracking log, a QR code for guided exercise videos, and an alert section for warning signs to support proactive pharmacist–patient engagement and ongoing safety surveillance.

To translate the conceptual “What–How–When” framework of chondroprotection into clinical practice, the CHONDROMOVING tool was designed as a single-page, infographic-based brochure that synthesizes medication guidance and lifestyle recommendations using intuitive icons and succinct messaging tailored to varying health-literacy levels. As shown in the left panel of Figure 7 (“the What”), a focused schematic of the knee-joint therapeutic targets for prescription-grade glucosamine sulfate and high-purity chondroitin sulfate is provided, accompanied by a gastrointestinal icon and a brief safety advisory based on current monitoring recommendations [39,40]. The right panel (“the How/When”) uses drug, exercise, and clock icons to illustrate two evidence-based strategies: morning dosing timed to coincide with circadian peaks in matrix-degrading enzyme activity and post-dose joint mobilization (e.g., cycling)—approaches further shown to enhance synovial fluid mixing and advective transport into diarthrodial joints and increase intra-cartilage residence of therapeutic agents [229,247]. By integrating medication guidance and lifestyle strategies into a cohesive, visually engaging format (Figure 7), CHONDROMOVING translates complex theoretical principles into simplified actionable steps, fosters meaningful pharmacist–patient dialogue, and promotes the safe and effective use of DMOADs in routine community practice.

To support ongoing patient engagement and safety monitoring, the reverse side of the CHONDROMOVING brochure could feature a structured symptom-tracking table (e.g., “Date|Pain Score|GI Symptoms|Comments”) for patients to complete during the critical first 3–6 months of chondroprotective therapy. Incorporating a QR code provides immediate access to an online demonstration of joint mobilization exercises and a downloadable dosing calendar with automated reminder alerts. A highlighted “Warning Signs” callout should instruct patients to report emergent issues, such as worsening glycemic control or elevated hepatic enzymes, to their pharmacist. This enables community pharmacists to initiate timely laboratory evaluation or prescriber referral, particularly for those with renal or hepatic compromise, where DMOAD administration warrants careful surveillance [39,40]. By embedding these practical tools within a unified, patient-focused format, CHONDROMOVING reinforces the pivotal role of pharmacists as essential partners in OA care, enabling proactive patient monitoring, tailored education on pharmacological and non-pharmacological strategies, and continuous optimization of treatment plans.

By synthesizing intuitive visuals, pharmacological–lifestyle integration, and safety protocols into a single user-centric resource, CHONDROMOVING translates chondroprotective theory into practice, enhances adherence to chronotherapeutic dosing, and strengthens pharmacist–patient collaboration essential for optimized community OA management. However, educational outreach alone cannot secure durable change; achieving sustained improvements requires the formation of cross-sector partnerships and strategic alliances with clinicians, pharmaceutical scientists, physiotherapists, payers, and patient advocacy groups to co-develop multidisciplinary care pathways and enhance therapeutic synergies.

### 9.3. P4 Medicine in Chondroprotection: A Multidisciplinary, Collaborative Framework for OA Management

**Figure 8:**
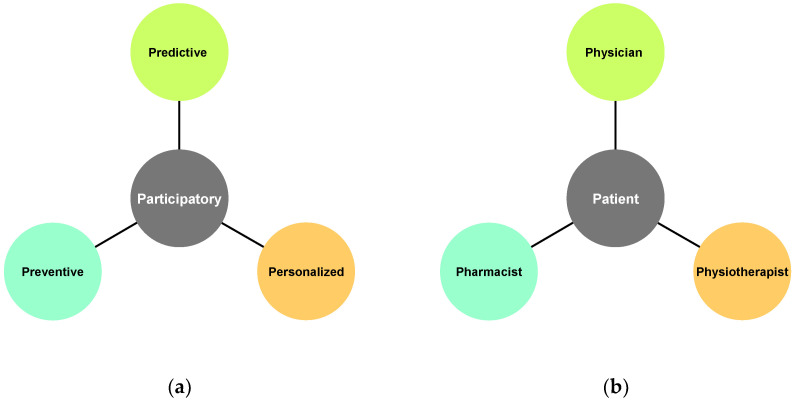
Dual representations of the “4P” paradigm in chronic disease management. (**a**) The four pillars of P4 medicine—Predictive, Preventive, Personalized, Participatory—characterized by risk stratification, early intervention, tailored therapy, and active patient involvement. (**b**) The corresponding 4P professional network—Physicians, Pharmacists, Physiotherapists, Patients—aligned with each pillar to underscore how multidisciplinary collaboration among these stakeholders drives a holistic, patient-centric care model in practice.

**Figure 9:**
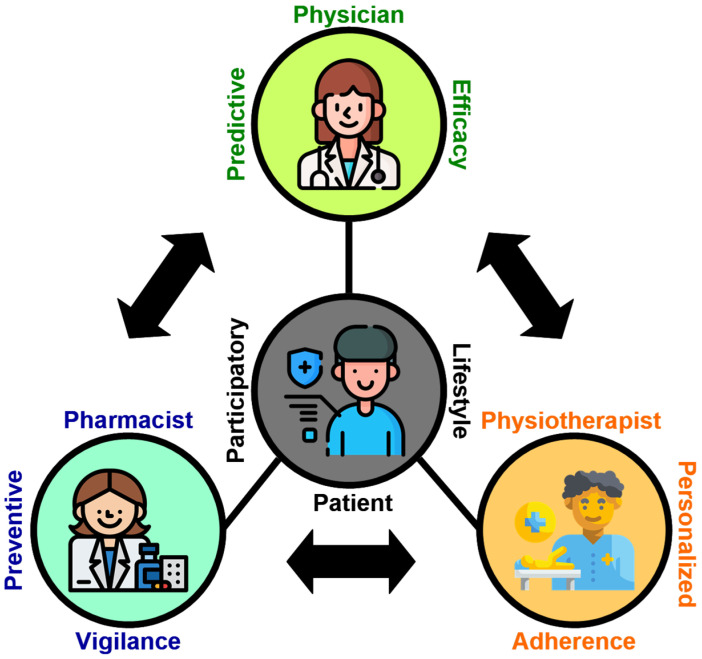
P4–4P Chondroprotection Framework for 21st-century OA management. This schematic aligns the four pillars of P4 medicine—Predictive, Preventive, Personalized, Participatory—with their respective professional stakeholders—Physicians, Pharmacists, Physiotherapists, Patients—to illustrate role-specific contributions and their synergistic interactions: Physicians → Predictive: risk stratification and initiation of disease-modifying osteoarthritis drugs (DMOADs); Pharmacists → Preventive: pharmacokinetic optimization and safety monitoring; Physiotherapists → Personalized: tailored biomechanical exercise regimens and lifestyle prescriptions; Patients → Participatory: self-management and real-time feedback. Bidirectional arrows denote continuous feedback loops—integrating real-world evidence, patient-reported outcomes, and interprofessional communication—to iteratively refine each pillar and optimize cartilage-preserving therapies. The 4P “dream team” represents a hypothetical “perfect” P4 multi-stakeholder collaboration in OA management, fostering an inclusive environment that drives truly patient-centered care and, ultimately, better clinical outcomes. Although not shown here for clarity, the complete framework further incorporates additional healthcare professionals—nurses, laboratory and pharmacy technicians, dietitians and nutritionists, occupational therapists, radiologists, clinical psychologists, social workers, patient educators and advocates, health coaches, exercise trainers, and caregivers—to deliver a truly comprehensive, multidisciplinary continuum of OA care.

P4 medicine—predictive, preventive, personalized, and participatory—constitutes a paradigm shift in OA management, yet its successful translation into chondroprotective practice requires a structured, multidisciplinary ecosystem [253,254,255,256,257,258,259,260,261,262]. Accordingly, the P4–4P Chondroprotection Framework (Figure 8 and Figure 9) was developed to integrate the four scientific pillars of P4 medicine with the four professional “Ps” essential to comprehensive OA care. Within this precision medicine model, (1) physicians employ genomic, imaging, and biochemical markers to stratify patient risk and initiate targeted DMOAD therapy; (2) pharmacists refine pharmacokinetic parameters and chronotherapeutic dosing, deliver individualized patient education, and conduct systematic safety monitoring; (3) physiotherapists design biomechanically informed exercise and lifestyle interventions—timed to coincide with peak synovial drug concentrations—to transiently thin the synovial unstirred layer and enhance advective transport of DMOADs into avascular cartilage; and (4) patients, together with caregivers, assume active roles in self-management, providing continuous feedback via digital adherence and outcome-tracking tools. This section first provides representative multi-stakeholder perspectives on current OA care gaps (see Table 2 for details), followed by a detailed description of the operational elements of the P4–4P Framework required to achieve precision-driven cartilage-preserving outcomes.

Table 2 provides a comprehensive synthesis of qualitative findings from the OA literature, capturing the perspectives of physicians, pharmacists, physiotherapists, and patients to elucidate the key challenges and opportunities in care delivery. Each group offers distinct yet interrelated insights that collectively underscore the need for more integrated and patient-centered approaches to OA management. Physicians, for example, describe a largely minimalist, reactive, and symptom-driven approach, often relying on basic analgesia and delayed escalation of care until the disease progresses to a more advanced or “crippling” stage [149,150,156]. The prevailing perception and narrative that OA is an inevitable consequence of aging further contributes to the minimization of early joint symptoms. Time constraints are commonly cited as barriers to delivering comprehensive education, while many clinicians also report limited therapeutic options and a lack of access to specialized OA resources [149,156]. Together, these findings underscore the inadequacy of relying predominantly on NSAIDs for OA symptom relief and call for comprehensive physician education in early OA detection, broader dissemination of evidence-based chondroprotective protocols, and systemic healthcare reforms designed to support proactive, patient-centered care—not merely from the initial manifestations of illness, but from the preclinical disease stages, before irreversible joint damage occurs [49].

Indeed, the intersection of deep-rooted clinical practices and societal narratives around aging creates a self-perpetuating gap in which early pathological changes remain invisible, delaying patient help-seeking and systemic intervention while perpetuating the silent progression of OA. Given its limited repair capacity [47], cartilage degradation is a critical initial event that fundamentally drives the development and progression of OA. Once this condition becomes clinically evident and is diagnosed, the chance of early intervention is often lost, leading to irreversible gradual progression of ‘early onset’ OA, which ultimately results in significant pain and disability [26]. Although joint deterioration typically accelerates with age, it occurs slowly, underscoring the need for early proactive management to enhance patient outcomes. However, the conventional “wait and see” or retardist approach—based on the assumption that OA warrants treatment only after symptoms become apparent—overlooks the reality that permanent structural damage to cartilage [22,49,149,158], including proteoglycan loss and collagen degradation, begins well before clinical signs are evident. This delayed onset creates a disconnect between early tissue changes and their association with the underlying disease, further exacerbated by longstanding misconceptions that hinder timely and effective OA prevention. This paradox of silent degeneration is magnified by cultural expectations of stoicism, gaps in health literacy, and a compartmentalized biomedical model—revealing the complex interplay between psychosocial attitudes and subclinical pathology that perpetuates delays in OA recognition and intervention.

Emerging evidence highlights the critical role of mechanical stress and low-grade inflammation in OA pathogenesis, challenging the prevailing paradigm and reconceptualizing the disease from a purely wear-driven “osteoarthrosis” to a complex inflammatory disease, “osteoarthritis”. This evolving perspective underscores that OA is a serious and debilitating condition with a burden comparable to that of RA [52,56,263,264,265,266,267]. Unlike autoimmune-driven inflammation in RA, OA is characterized by non-classical inflammatory processes at the synovium-cartilage junction (see Figure 1 for details). These processes, mediated by autocrine and paracrine signaling, involve unique cytokine profiles that contribute to joint degeneration, underscoring the severity of OA and its distinction from RA [47,48,49,50,51,52,53,54,56,117,176,267]. This shift in understanding underscores that OA is not an inevitable aspect of aging and highlights the critical importance of early intervention and comprehensive management strategies to prevent irreversible joint damage and improve patient outcomes.

However, a significant challenge remains: how can we effectively prevent the development of OA without a robust scientific framework that provides a comprehensive understanding of the chemical and biological processes governing cartilage metabolism and its etiopathological mechanisms? Despite significant advancements in modern medicine, this area remains poorly defined. Consequently, OA is often misunderstood, with widespread myths and misconceptions obscuring its true nature and impeding efforts to prevent and detect the disease in its early stages. A common misconception, as mentioned above, is that OA is an inevitable consequence of aging—a natural part of the aging process that cannot be avoided. This belief leads many individuals to overlook early signs and symptoms, ultimately delaying preventive measures [152,157,158,161,268]. OA is a multifactorial disease influenced by various modifiable risk factors, including excess body weight, insufficient physical activity, and other lifestyle-related determinants that collectively affect disease onset and progression [26,269]. Additionally, failure to distinguish between post-traumatic and idiopathic (non-traumatic) OA further complicates the clinical landscape, as these forms of the disease exhibit distinct pathophysiological mechanisms and may require different therapeutic approaches [87]. Clarifying these distinctions and addressing modifiable risk factors are essential for developing personalized treatment strategies and effective public health initiatives in this area.

A new model for understanding OA based on multiple phenotype/endotype-guided approaches has recently emerged to address disease heterogeneity, identify risk factors, and patient stratification for the pathogenesis and progression of this complex joint condition [172,269]. While strategies to manage bad days and symptom exacerbations remain important, particularly for those facing daily activity limitations, the optimal approach lies in pre-symptomatic intervention during the early trajectory of the condition before clinical onset. Advancing this paradigm demands a transformative reorientation of health policy and public discourse to recalibrate prevailing narratives and increase awareness of the progressive nature of the disease. Despite its increasing prevalence, OA is often disregarded and marginalized in public discourse, possibly because it does not directly cause death, although it significantly affects morbidity and quality of life [25,26,153]. The considerable morbidity and mortality associated with this common condition are often underestimated due to its insidious progression and widespread misconceptions regarding its inevitability with aging. This underscores the need for a nuanced health education approach that increases the perceived severity of OA to that of critical illnesses, such as CVD, promoting early intervention strategies without resorting to fear-based tactics. Confronting the fatalistic view of OA as an unavoidable consequence of aging is crucial, as it delays timely medical engagement, reduces the chances of non-surgical interventions, and ultimately leads to a cascade of comorbidities that worsen the condition [25,26]. As shown in Table 2, this issue is exacerbated by systemic barriers to timely diagnosis and access to care [3,22,145,149,150,154,156,158]. Additionally, it is imperative to critically examine the techno-optimism associated with joint prostheses—both among patients with OA and, notably, among healthcare providers—who often expedite total knee or hip arthroplasty while underutilizing rigorous, evidence-based non-surgical interventions [145,149,158,270,271]. The principal insight from Table 2 underscores the imperative of a prevention-first paradigm—detecting OA at its incipient, manageable, and modifiable stage rather than waiting for an entrenched disease state that resists both medical intervention and patient self-management. This intersection of clinical inertia, sociocultural beliefs, system-level structural healthcare gaps, and fragmented care delivery illustrates OA as a paradigmatic example of how biopsychosocial and biomedical complexities converge—necessitating proactive, interdisciplinary approaches to disrupt the trajectory toward irreversible joint deterioration.

Building on our earlier discussion, public health campaigns should emphasize that OA is not an unavoidable outcome of aging but rather a condition that can be both prevented and effectively managed. Furthermore, these initiatives must advocate precise patient stratification, acknowledging the diverse and distinct pheno-endotypes of OA [139,173,272,273,274,275,276], which is crucial for tailored treatment and improvement of outcomes. OA is a progressive, multifaceted disease, with each subtype exhibiting unique features and variability that can differ significantly among patients [275,276]. For that reason, OA should be viewed as a common endpoint rather than a singular final pathway, reflecting that while the disease manifests similarly in its advanced form, its development can arise from a variety of unique etiological factors, distinct underlying processes, and specific subtypes, including post-traumatic, metabolic, post-menopausal, and age-associated phenotypes. Challenging misconceptions and raising public awareness about preventable factors of OA, along with the critical role of early intervention, can significantly reduce the burden of this chronic condition. In this regard, OA narratives are frequently characterized by catastrophic pain anticipation and kinesiophobic avoidance, wherein individuals preemptively restrict their movement out of fear that any activity will exacerbate joint deterioration [87]. Such fatalistic and resigned attitudes—manifesting as therapeutic procrastination, help-seeking delay, and learned helplessness—underscore a pervasive sense that nothing can be done, ultimately undermining proactive self-management and accelerating functional decline. By reshaping the narrative surrounding OA, individuals can be empowered to take proactive measures to safeguard their joint health long before disease progression and irreversible damage occur [277]. Therefore, addressing OA effectively demands interventions targeting maladaptive beliefs and behavioral patterns as much as it targets joint pathology, reaffirming OA as predominantly a psychosocial and behavioral condition rather than solely a biomedical one [4].

Building on this broader psychosocial approach, pharmacists emerge as key players in bridging the gap between behavioral support and medical management. Pharmacists, as highly accessible healthcare providers, identified community pharmacies as critical points of entry for broader OA care (Table 2). They reported successfully engaging individuals who otherwise might not have access to traditional care settings and emphasized their expanding role in ensuring medication safety and educating patients on proper drug use. According to McLachlan et al. [20], almost three out of five patients diagnosed with OA do not actively seek medical care. A proactive engagement strategy, wherein pharmacists initiate conversations about OA management, appears to foster trust and has the potential to bridge gaps in patient knowledge [7,22,23]. Additionally, pharmacists see an evolving role for their profession—transitioning from medication dispensers to active participants in lifestyle and disease management support—which could complement the more reactive, symptom-focused approaches commonly observed among physicians. However, high levels of health literacy are critical for patients to translate clinical recommendations into meaningful self-care behaviors. In community pharmacies, this means distilling the pharmacological intricacies of DMOADs into succinct patient-centered messaging.

Yet, this need for clarity underscores a broader systemic challenge: many patients continue to report dissatisfaction with the quality and comprehensibility of the information provided across healthcare providers, highlighting persistent gaps in communication that compromise effective OA management [22,141,142,144,148,149,150,156,157,158]. Despite expressing confidence in their physicians, patients frequently perceive a lack of comprehensive discussion regarding OA pathophysiology, available treatment options, and therapeutic approaches for sustained, long-term disease control. As detailed in Table 2, many patients described healthcare engagement as predominantly reactive, requiring them to initiate consultations, which resulted in significant frustration and uncertainty [144]. Time constraints in clinical settings further hinder comprehensive discussions, leaving patients uninformed and unsupported. The terminology clinicians employ can markedly influence the way patients perceive their condition; for instance, characterizing OA as “wear and tear” often triggers negative assumptions, perpetuating the belief that the disease is an inevitable and untreatable consequence of aging. Furthermore, the absence of structured, clinician-led education exacerbates reliance on the internet, where the quality and accuracy of information can be inconsistent [278]. To improve patient satisfaction and engagement, healthcare professionals should prioritize clear, patient-centered communication, offer accessible educational resources, and adopt individualized approaches that address both informational and emotional needs in OA management. Given the substantial evidence that OA frequently leads to anxiety and depression [4,25], failure to address the psychological sequelae of OA not only undermines treatment efficacy, but also violates the principles of holistic care. Further, neglecting the psychological burden of OA transcends mere omission and rises to the level of malpractice. Only by coupling transparent communication with emotional support can we truly empower patients and mitigate the anxiety and depression that frequently accompany this misunderstood “wear-and-tear” disease.

Patient perspectives, as illustrated in Table 2, highlight widespread dissatisfaction regarding the adequacy and clarity of OA-related information provided by healthcare professionals in clinical encounters. As discussed earlier, many report that their OA diagnosis was minimized to “wear and tear”, without meaningful discussion of the underlying pathophysiology or its long-term implications. Consultations are often described as impersonal and lacking in follow-up or tailored guidance, prompting patients to seek information independently, predominantly online, thereby risking misinformation and further confusion [144,149]. Beyond informational deficits, patients articulate significant emotional distress, including feelings of resignation, the burden of chronic joint pain, and fears of eroded independence, which can foster fatalistic attitudes toward OA progression [141,146,148,152]. However, amidst these challenges, a subset of patients demonstrates resilience by proactively exploring adjunctive strategies, including nutraceuticals, complementary therapies, and tailored exercise regimens, in an effort to assert control over their OA management. Importantly, patients identify pharmacists as highly accessible and reliable sources of OA information, valuing their regular frontline (‘coal-face’) interactions and their demonstrable expertise in disease management [22].

In the current digital era, in which individuals increasingly rely on online search engines, AI-driven chatbots, and social media for health information, the risk of encountering misleading or inaccurate OA advice is significant [278]. Community pharmacists can help counteract this “infodemic” by recommending and disseminating a vetted compendium of digital tools—ranging from accredited symptom-tracking applications and secure teleconsultation services to authoritative guideline repositories (OARSI, EULAR, ACR)—and by establishing strategic alliances with leading patient advocacy organizations (e.g., OAAA, OAFI, Arthritis Foundation, Arthritis Australia) to enhance resource credibility and patient engagement [91,92,93]. During consultations, pharmacists should provide hands-on demonstrations and concise take-home guides to reinforce digital health literacy and patient confidence. Structured patient education on evaluating online content—focusing on publication date, source credibility, and peer-review status—enables the critical appraisal of AI-generated and social media health claims. Furthermore, integrating wearable sensors and mobile health applications to monitor joint mobility, pain levels, and exercise adherence can generate real-time data for personalized counseling. Embedding CHONDROMOVING-style interactive modules into these smartphone apps and digital platforms, delivering “What–How–When” guidance, timely medication reminders, exercise demonstrations, and symptom logging, can further enhance patient engagement and self-management. This hybrid model, which combines curated digital tools, critical evaluation training, and objective activity monitoring, may empower patients to make informed decisions regarding chondroprotective pharmacotherapy-oral drugs/supplements, physical therapy, and lifestyle integration, and reinforce the position of community pharmacists as trusted partners in both face-to-face and virtual OA care.

In addition, pharmacists can leverage concise, relatable, and patient-friendly analogies to correct the common misconception that OA is merely an inevitable consequence of aging. One effective illustration is the clinical paradox—“both knees are the same age, yet only one hurts”—which instantly conveys that joint-specific biological mechanisms drive symptom development rather than chronological senescence alone. In practice, a pharmacist might explain, “If OA were simply ‘wear and tear’ from aging, both knees would be equally affected; the fact that only one hurts indicates localized stressors or early joint changes”. Framing the discussion in this way opens the door to explain how aberrant biomechanical loading, joint-specific injury history, and tissue-level inflammatory processes contribute to cartilage degeneration [47,48,49,50,51,52,53,117]. By deploying a mere analogy—a straightforward, illustrative comparison—to refute the “wear and tear” myth, pharmacists empower patients to understand the rationale for targeted interventions, such as chronotherapeutic DMOAD strategies that address the underlying pathology (endotype) rather than simply mask pain. This approach not only enhances patient understanding and engagement but also supports more proactive and preventive management of OA. Such enhanced comprehension strengthens confidence in pharmacist-led counseling and establishes community pharmacies as pivotal access points for individuals in the early stages of OA to receive appropriate care.

Building on this trust, pharmacies represent a critical—yet often underutilized—point of contact for individuals experiencing early symptoms of OA. Many people seek OTC analgesics and anti-inflammatory medications at pharmacies before consulting a healthcare provider because of their accessibility, proximity, and absence of appointment requirements. This common behavior positions pharmacists as key frontline contributors to pharmacovigilance efforts in OA care. Although pharmacists lack diagnostic tools to definitively identify this heterogeneous multifactorial condition [20,22,279], they can play a pivotal role in early identification by recognizing patterns of recurrent pain medication purchases and providing referrals to healthcare providers for proper evaluation and management. Integrating pharmacies into a collaborative healthcare framework could enhance the early detection of OA, optimize patient education, and support pharmacovigilance by monitoring medication usage patterns. However, this approach requires additional training for pharmacists and improved communication pathways between pharmacies and healthcare centers to ensure continuity of care and accurate diagnosis.

Although community pharmacist-led interventions for OA have demonstrated efficacy in improving chronic pain management [280,281], they show only minimal effects on physical function, health-related quality of life, and depressive symptoms, underscoring the need to integrate additional disciplines into the care continuum. Physiotherapists contribute a holistic perspective to OA management by integrating biomechanical insights with behavioral strategies. As detailed in Table 2, they emphasized the importance of explaining OA pathophysiology to foster a realistic understanding of the disease process [145,160]. Despite this educational emphasis, many express skepticism about the potential for long-term structural modification, anticipating that progressive joint deterioration culminates in surgical intervention. Nonetheless, physiotherapists consistently advocate patient empowerment through tailored exercise programs and lifestyle modifications, particularly weight management, as cornerstone strategies for preserving function and slowing disease progression. Significant challenges were observed in sustaining long-term adherence to prescribed exercise regimens, largely attributed to psychological barriers, fear-avoidance behavior, and limited follow-up resources (refer to Table 2 for further detail). These insights highlight the necessity for innovative adherence-support mechanisms and sustained multidisciplinary collaboration to reinforce behavioral changes over the chronic course of OA.

As depicted in Figure 9, the P4–4P Chondroprotection Framework constitutes an integrated multidimensional model in which the four scientific pillars of P4 medicine—Predictive, Preventive, Personalized, and Participatory—are embodied by a distinct healthcare stakeholder group. Within this theoretically grounded interdisciplinary framework, physicians support the “Predictive” pillar through multi-omics approaches—including genomic, epigenomic, and metabolomic profiling—combined with advanced imaging and biochemical biomarkers to stratify OA risk and initiate targeted DMOAD therapy at the earliest subclinical stage [118,119,120]. Pharmacists reinforce the “Preventive” pillar by refining medication selection, optimizing pharmacokinetic profiles and chronotherapeutic regimens, delivering personalized patient education, and systematically monitoring safety to prevent adverse events. Physiotherapists embody the “Personalized” pillar by prescribing biomechanically informed exercise and lifestyle interventions—timed to exploit synovial fluid dynamics—to enhance intra-cartilage drug delivery, and sustain joint function. Finally, patients represent the “Participatory” dimension, actively co-managing their treatment via real-time adherence reporting and outcome feedback through digital health platforms. Reciprocal feedback mechanisms interlink each professional role with the P4 components (Figure 9), driving iterative optimization of risk-stratification algorithms, preventive modalities, personalized strategies, and participatory engagement. Overall, the P4–4P Framework likely provides a precision-driven, cartilage-protective paradigm for OA management that transcends traditional fragmented care [144].

A growing body of evidence demonstrates the feasibility and impact of structured pharmacist-led OA services within community settings. In the PharmOA study, Babatunde et al. [282] co-developed a five-phase intervention comprising systematic evidence synthesis, stakeholder interviews, co-design of risk stratification tools and chronotherapeutic guides, and an implementation pilot that achieved high pharmacist and patient acceptability, improved medication adherence tracking, and stream-lined referral pathways. Similarly, Simkins et al. [23] conducted a multi-method evaluation of an extended OA service model in community pharmacies, confirming that pharmacists can deliver assessments, medication management, and self-care support, albeit with workload and health-record access challenges to address. A cluster-randomized trial by Marra et al. [10] further revealed that pharmacist-initiated multidisciplinary interventions significantly reduced pain and enhanced physical function and quality of life compared with usual care. Notably, Artro 360 integrates early diagnosis, risk stratification, preventive and rehabilitative pathways, and seamless coordination among pharmacists, physicians, nurses, and patient organizations to optimize resource use and improve clinical outcomes, with preliminary health-economic analyses forecasting significant annual savings alongside enhanced patient satisfaction and guideline-adherent care [42]. Together, these studies underscore the pivotal role of community pharmacists as accessible, patient-centered hubs that implement the P4–4P Chondroprotection Framework, delivering preventive education, tailoring individualized dosing regimens, and facilitating participatory self-management in OA care, while concurrently broadening their contributions to guideline-based management and generating real-world evidence to iteratively refine chondroprotective strategies. To translate these promising pharmacist-led models into scalable, sustainable practices, stakeholders across the translational continuum—including basic and implementation researchers, academics, policymakers, regulatory authorities, payers, frontline clinicians, pharmaceutical and biotech companies, professional societies, and patient advocacy organizations—must collaborate to coordinate funding mechanisms, regulatory frameworks, and care pathways, thereby closing persistent evidence gaps and ensuring equitable, high-quality delivery of P4–4P chondroprotective interventions.

## 10. Summary, Conclusions, and Recommendations

OA remains a leading cause of chronic pain and functional limitation worldwide, yet traditional care models—characterized by reactive, symptom-focused interventions—have failed to arrest its progressive, cartilage-destroying trajectory [3,26]. In this paper, I have (1) delineated the pharmacological basis and mechanistic rationale for chondroprotection via DMOADs; (2) demonstrated the critical need to optimize their chronotherapeutic timing, delivery methods, and patient engagement through the “What–How–When” framework; and (3) introduced an integrated P4–4P Chondroprotection Framework that aligns predictive, preventive, personalized, and participatory medicine pillars with the complementary expertise and distinct contributions of physicians, pharmacists, physiotherapists, and patients. I further illustrated how innovative patient-centered tools, such as the CHONDROMOVING brochure, can translate complex theory into actionable practice, and highlighted empirical models (e.g., PharmOA, Artro 360) [42,282] that validate the expanding role of community pharmacists in delivering evidence-based OA services. Currently, there is a lack of published data from high- and low-to-middle-income countries on pharmacist-led holistic chondroprotective interventions aligned with the proposed P4–4P framework. As this field remains in an early conceptual stage, future research should prioritize inclusive multicenter investigations that address regional differences in OA burden, healthcare access, and implementation feasibility.

Moving forward, stakeholders must develop and disseminate rigorous, evidence-based DMOAD clinical guidelines detailing pharmaceutical-grade agent selection, dosing regimens, chronotherapy principles, and safety surveillance, and establish formal, multidisciplinary OA care pathways that integrate physicians, pharmacists, and physiotherapists. Concurrently, structured community-based programs should be implemented to provide comprehensive risk assessment, chronotherapeutic counseling, adherence support, and lifestyle coaching. Equally critical is embedding chondroprotection, chronotherapy, and inter-professional collaboration into the curricula of medicine, pharmacy, and allied health programs [12]. Concurrently, researchers should conduct pragmatic, practice-based trials to evaluate the long-term structural and functional benefits of combined pharmacological and exercise-timed regimens, while educators and regulators should work in concert to equip practitioners and reshape healthcare incentives toward holistic cartilage-preserving OA management.

To bring this ambition to fruition, high-quality, large-scale, double-blind randomized controlled trials (RCTs) should be prioritized to assess the long-term structural preservation, functional, and quality of life outcomes of synchronized DMOAD and exercise-timed interventions [95], with robust real-world evidence data to iteratively refine best practices. By realigning research, clinical protocols, education, and policy around the comprehensive P4–4P Chondroprotection Framework, the OA care community can shift from symptom management toward true disease modification—preserving joint integrity and significantly enhancing patient outcomes.

## Figures and Tables

**Table 1:** Overview of representative OA therapies, categorized by pharmacologic class. † Investigational or preclinical only. Data adapted from references [29,30,31,32,34,37,38,39,41,44,45,46,50,52,57,58,59,60,61,65,70,71,72,74,75,77,78,79,80,81,82,83,84,85].

Category	Sub-Class	Example Agents
Rapid symptomatic relief	Analgesics	Paracetamol (acetaminophen)
	NSAIDs	Ibuprofen, diclofenac, celecoxib, etoricoxib
	SNRIs	Duloxetine
	Opioids	Tramadol, oxycodone, buprenorphine, fentanyl
	Intra-articular corticosteroids	Triamcinolone acetonide, nethylprednisolone acetate, betamethasone sodium phosphate
Slow-acting medications	Chondroprotective agents	Glucosamine sulfate, chondroitin sulfate, diacerein, avocado/soybean unsaponifiables (ASU)
	Disease/structure-modifying agents †	Lorecivivint (SM04690), sprifermin (rhFGF18), MIV-711, selective inhibitors of MMP-13 and ADAMTS-5, small-molecule ECM modulators
	Intra-articular viscosupplementation	Sodium hyaluronate, hylan G-F 20 (Synvisc), high-molecular-weight and cross-linked formulations Polyethylene glycol-based hydrogels, carboxymethylcellulose, chondroitin sulfate derivatives Recombinant lubricin (PRG4), phospholipid-based lubricants, synthetic glycosaminoglycan mimetics
Regenerative medicine and tissue engineering †	Platelet-rich plasma	Autologous PRP formulations varying in platelet and leukocyte concentrations (e.g., leukocyte-rich vs. leukocyte-poor PRP); classification and standardization remain under active investigation to improve reproducibility and clinical translation
	MSC-derived exosomes/Stem cells	iPSC-derived MSCs, MSC exosomes
	Cell-based and gene therapies	Gene-edited chondrocytes, engineered MSCs
	Biomaterial scaffolds	Injectable hydrogels, nanoparticle carriers

**Table 2:** Multi-stakeholder perspectives on OA management. Representative qualitative quotations from physicians, pharmacists, physiotherapists, and patients addressing challenges in patient engagement, disease education, medication safety, exercise adherence, and treatment uncertainty. Citations are provided for each quotation.

Perspective	Theme	Quote	Reference
Physician	Minimalist, reactive care—a stepwise but limited approach to managing OA	*If somebody came and saw me with…knee pain, I’d probably tell them to take some paracetamol and go away. And if they came back and saw me again…and said it was still painful, then I might send them for an x-ray. And then if that came back and said osteoarthritis I would…say, “Well yes it’s osteoarthritis, but there isn’t very much we can do about it at the moment.*	[149]
	Viewing OA as part of natural aging	*(Knee osteoarthritis) is part of getting older, it’s normal really, not normal, but it makes sense as it is part of the natural evolution.*	[150]
	Age-related minimization—normalizing OA as an inevitable part of aging	*Because it’s a condition that’s associated with aging…both the patients and the health care professionals will tend to say, “Oh it’s just a part of getting old,” so that they will tend to minimize it. …And health care professionals, I think, just tend to leave them to get on with it…you are often told, “Oh my GP just says well, you know, it’s just something you have to learn to live with.*	[149]
	Reflecting on explanatory language	*I’ve been guilty of talking about osteoarthritis as the wear and tear type of arthritis … it does challenge some of the ways in which we explain osteoarthritis to people.*	[159]
	Time constraints in delivering care	*The bigger issue is, I feel I don’t have enough time to really give it in a way that I’m completely satisfied with.*	[156]
	Delayed intervention—early symptoms are minimized until the condition becomes severe	*I think it’s the amount of time people have to actually wait before anybody does anything. You have to get to a certain level with your arthritis before anybody is actually going to listen to you. Just aches and pains and a small amount shown on an x-ray of arthritis, is probably just thought nothing of. You have to wait until it gets to that really crippling stage almost before anybody does listen to you.*	[149]
	Underfunding and research gaps in OA	*There are very little studies done (…) there might be small teams of researchers working on osteoarthritis in various countries but there are no significant funds allocated to osteoarthritis. I recognize that if we want people to work till they are 70, we will need to do something against osteoarthritis!*	[150]
	Lack of adequate information and comfort with OA	*We, patients and physicians, are in the dark! (…) We are not comfortable with this pathology. (…) We really ought to know what are the impacts and the procedures according to the patients’ profiles and risks.*	[150]
	Oversimplification—providing rudimentary, insufficient patient education	*But quite often I only give them a very basic explanation of what osteoarthritis is…rudimentary sort of wear and tear, degenerative explanation, which of course is only part of the story. …I’m trying to think if I use any patient information leaflets, and I don’t regularly do that for osteoarthritis actually… there is a lack of information I’d say.*	[149]
	Need for patient education in self-management	*If somebody had time and could concentrate on that person and educate them more about it then perhaps that person could self-manage their arthritis better.*	[149]
	Enhancing patient confidence via exercise demonstration	*A physio showing us a few exercises…that would be a very good thing… for us to feel a bit more confident.*	[156]
	Challenges in motivating patients	*The problem is how do you actually get people to do this stuff…how do you tell them what the right thing to do is?*	[156]
	Frustration with non-adherence	*There are a lot of patients who are lazy…won’t carry out instructions and the recommendation to exercise.*	[156]
	Hope for regenerative treatments	*We are all dreaming of a product which would rebuild the cartilage just by injecting it into the joints.*	[150]
	Call for comprehensive education—empowering patients through better information and support for self-management	*I think just better education around everything, around the nature of the disease, what it involves, about what the treatments are, and about not just sort of tending to think, “Oh well I need a knee replacement and…that’s it. And I’m not going to get any better until I get one.”*	[149]
Pharmacist	Pharmacies as accessible entry points to healthcare	*I thought it was good being done in pharmacy, because we see a lot of people who don’t access any healthcare. But they’ll walk into a pharmacy and look at the glucosamine or something. So there were a few people like that, that I kind of grabbed, who I know hadn’t talked to any health professional at all about OA, but it was quite clear they would benefit from an intervention of some description. So it was quite nice to be able to pull them into healthcare a bit more*.	[22]
	Mixed perceptions of intervention deliverables	*[But] they thought it was a wonder drug we had on offer… and some people were like, “well I can’t rub information on my knee”.*	[22]
	Ensuring safe, effective medication use and patient education	*But then, if it’s about medicines, it’s making sure that they’re safe, and effective, and optimised, I guess, is my biggest thing. So not only about recommending the right medicine for that person, but it’s also about educating them on how to take it safely.*	[23]
	Proactive engagement increases patient trust	*When we approached them about knee arthritis, they never thought we would be the professional that could talk about it… I think the fact that we actually brought it up, for them, it’s really an important engagement strategy.*	[22]
	Supporting lifestyle interventions	*And I think we’re quite well positioned for like lifestyle interventions if somebody was interested. And potentially like they wanted to talk through managing other health conditions and, if there was any support, they could maybe have with like, diet, exercise… we’re quite well positioned to do that.*	[23]
	Tangible resources enhance engagement and follow-up	*[Patients] were very happy to walk away with something physically from that interaction… I had someone ask me something, I led with a leading question, they were able to give me a lot more information—it set the scene for a more in-depth discussion, and I found that physically being able to hand them something at the end of that interaction was kind of a take-home for them. Every person I spoke to was very happy to receive something like that, that they could take home and look at in their own time. And then I always said, “look, if you have any more questions, come back and see me. That’s what we’re here for”.*	[22]
	Evolution of pharmacy practice toward proactive care	*This is definitely where we’re heading in terms of our pharmacy profession… Are we asking the right questions, are we actually giving them the solutions that they need, and are we telling them something they don’t know about? Those things are big wins for us.*	[22]
Physiotherapist	Explaining OA pathophysiology	*When I start to explain OA, is about ‘Over time, basically the joints start to wear down, usually from loading.’ It can be other factors but most of the time is from loading.*	[160]
	Pessimism regarding long-term modification of OA	*The arthritic degeneration is going to continue to ‘rumble on’ ‘pretty much’ whatever we do and so, ultimately, if it was reviewed in three years, I feel quite sure that the arthritic changes would be worse, irrelevant of what we do.*	[145]
	Expectation of inevitable progression leading to surgery	*My guess is that, regrettably, long term her knee is only going to get worse, assuming that it is arthritic and, really, a knee replacement is the answer.*	[145]
	Empowering patients to take control of their care	*Obviously, I would say to them, ‘it’s your life.’ I mean, ‘but we’re trying to improve and help you manage your lifestyle, manage your pain and obviously, this is all part of it. If you feel that you want to come and get on board and help yourself, then these will help you. If you don’t, then it’s just a waste of time.*	[145]
	Patient responsibility with professional support	*I think it’s the Physio’s responsibility to motivate the patient and explain why they’re doing it, but, ‘in the end,’ it’s their responsibility to do the actual exercise. So, once you’ve presented the case and presented the information, and the reasoning for it, and what you’re expecting them to achieve and by what time and when; it’s totally up to them to do that, but with your support, initially.*	[145]
	Promoting joint movement for optimal cartilage health	*This idea of this arthritis, one of the things I like to do with the patients is to make sure (that) they’ve got as full a range of motion as possible, with the idea of trying to lubricate the joint and to get the synovial fluid to give its nutrition through to the cartilage, and I think one of the ways of doing that is moving the knee through that full range of motion and I think there’s a danger, with patients, if they’re not…if they’re in pain, or getting stiffness (such) that they tend to avoid the movement and then they just get stiffer and if they’re getting stiffer, they’re not getting that lubrication and they’re not feeding their cartilage to try and keep it as healthy.*	[145]
	Integrating weight loss into exercise prescription	*If weight-loss is a contributing factor then you’re talking about referral to a dietician—plus talk to them about non-weight-bearing cardio exercises… I like cycling if the joint is happy for that bending and extension if that’s not irritable. I’d probably start with cycling because it’s less weight-bearing and then work on slowly progressing that.*	[160]
	Importance of adherence for treatment efficacy	*Of course, if they’re not complying with that and they’re not doing the exercises as you’ve prescribed them, they won’t be as effective in the same way as a doctor prescribes you to take four painkillers a day and you only take two, you’re not going to be as comfortable as if you’d taken the four.*	[145]
	Patient heterogeneity in adopting behavioral changes	*Some patients are very…you know, they’ll come along; they will listen to the reason why they need to change their activity and will readily change. Some patients are just so resistant to change. I mean, I’m thinking of one lady, at the moment and there’s such psychological barriers there and I don’t find I’ve got the time or the resources to deal with that.*	[145]
	Long-term adherence challenges	*The main challenge we find is the actual compliance—the patients doing their exercises—How do we get this going on a long-term basis? How can we maintain? Yes, they’ve come and seen us in the last three months and improved a lot, but we can’t keep seeing them for the next five, six, 10 years.*	[160]
	Barriers in follow-up due to resource constraints	*My frustration is not being able to follow people up sufficiently to actually identify those [nonadherent patients] and this is because of restraints of time, and in the private sector, restraints of people, of the cost for the individual patient.*	[145]
Patient	Sudden, visceral onset of structural joint breakdown in everyday life	*“One day I was walking good, the next day bang … the bone was catching on bone. You can feel it actually grinding”.*	[158]
	Insufficient explanation—diagnosis is trivialized and not fully explained	*I don’t think you ever get told what it is, or why you’ve got it, except that well it’s just wear and tear, and that’s as far as you go.*	[149]
	Stereotypical “wear and tear” explanation	*They always say same thing: wear and tear, you know, you’re getting older.*	[157]
	Dismissive medical encounters	*So I go to the doctor and all he just simply done was put his hand on my knee, he said ‘move your leg…you are getting old you’ve got rheumatism.’ You see that was it I didn’t take any more notice of it [the knee pain].*	[141]
	Impersonal consultations—technology interferes with face-to-face connection	*His face is hidden by the computer. His eyes are on the computer, I can’* *t see the way he is looking. The consultations could almost be done over the phone.*	[144]
	Dismissive communication—patients feel unheard and brushed aside	*They don’* *t understand us, they don’* *t listen to us. They are very cool. They are tense. They don’* *t take enough time. You have to get them to talk, I’* *m afraid if you don’* *t ask, they don’* *t say anything. They tend to avoid the question. They don’* *t want to say too much. Take this and that, with no explanations. They have no cure. They are helpess. You just have to put up with it and that’* *s that. You dare not ask (for information). They don’* *t really like people asking questions. He made me feel I was being a nuisance. I told him about newspaper articles about a new treatment, and he just waved it away.*	[144]
	Abrupt, impersonal delivery—leading patients to seek information on their own	*I got a phone call to say, “I’ve got some bad news, you’ve got arthritis in both hips.” …He didn’t say, “Come and see me and we’ll talk about it.” It was, “goodbye.” …I think he said, “You’ve got marked arthritis in both hips,” and I didn’t really understand what that meant. So I had to go on the internet and have a look and see what it was all about.*	[149]
	Necessity for patient advocacy and proactive communication	*You always have to take the initiative. (I have to say) I am suffering terribly, please give me something. You always have to ask. A doctor can’* *t know everything. I feel that (the doctor) was right and courageous when he said that he couldn’* *t give me clear information, and he sent me to someone who could.*	[144]
	Lack of guidance—insufficient dietary and lifestyle advice provided by clinicians	*When you are diagnosed with it, you are not given enough information, now I have never been told at all about anything to do with certain foods not to eat. But I have found out since there are certain foods that you shouldn’t touch…that was picked up on the internet by my son, but I mean I have never been told by a doctor to cut out anything that would aggravate arthritis.*	[149]
	Perceiving OA as a slow, cumulative condition	*Stroke gets front-page news, but people don’t die of OA all at once. Your life is whittled away.*	[151]
	Fatalistic view of OA progression	*I don’t think it would improve. It may stay the same, but I would expect it to get worse… you can’t change osteoarthritis.*	[157]
	Resignation to disease inevitability	*There is nothing that can be done about the OA; therefore, I do nothing.*	[146]
	Loss of independence and living in constant misery	*I describe the pain as living with misery … it makes you feel miserable … makes you feel that if this is all … it is for the rest of your life … and I have always been a really independent person and now I’ve got to be dependent on someone else … and that’s been killing me.*	[152]
	Emotional toll and chronicity of OA	*Living with arthritis … it’s a living nightmare … I wouldn’t wish it on anybody … It’s not like having a broken arm … it hurts at the time … but it mends … arthritis to me it does not … the last 15 years … nobody actually said oh! You will be cured … so it’s ongoing … and I perceive it … it only will get worse … It limits me in what I can do … which is frustrating … and sometimes all those worries about the future … really get me down …*	[152]
	Embodied singularization of OA experience as counter-narrative to standardized disease taxonomies/Personalized disease identity (phenotypes)	*“My osteoarthritis is not the same as the millions of others”.*	[161]
	Emotional distress and loss of identity	*I was extremely unhappy with myself. I couldn’t work as hard as before, and I just could not understand why. It was one of the hardest things, to accept myself as what I had become.*	[146]
	Reduced autonomy and emotional impact	*I’m very upset with myself cos [because], you know, when you’re used to being mobile and able to do things for yourself, now you have to depend on people to do it, it’s not very nice is it?*	[141]
	Feeling neglected compared to other arthritis types	*As a person with OA I felt like an orphan in the arthritis world. I am thrilled that medications and biologics are available for folks with [rheumatoid arthritis]. For many it has changed their lives. But as a person with OA, I am frustrated that research was not there for me and others like me.*	[151]
	Skepticism about medication as a cure	*I’m not keen to take things because they’re not going to cure it, and I mean to hide it is not strictly a good idea because you do things and it makes it worse.*	[142]
	Limited benefit of analgesics versus ongoing degeneration	*All it’s really doing is taking the pain away a little bit. But the joint continues to deteriorate, the pain gets worse.*	[157]
	Mixed efficacy of painkillers and self-management	*I am the one who knows when it hurts too much. If it is unbearable, I take painkillers. But (…) the painkiller I take gives me stomach problems although it really works on the pain. If I do feel pain but it’s not too serious I take paracetamol. (…) I deal with it according to the pain. He (my doctor) gave me Diclofenac but it has never really worked.*	[150]
	Medication avoidance/Self-reliance in pain management	*I don’t want too many tablets in me… I can try and bear pain myself.*	[147]
	Low medication reliance/Acceptance of pain	*I am not a one for taking a lot of tablets. I get a bit dubious, you know, so I just learnt to live with it for a bit.*	[141]
	Need for clear, detailed treatment information	*I would like someone to explain to me why and how the dose should be increased when there is a flare-up, and why it is decreased afterwards.”* *‘Why is it important to avoid getting too used to these drugs [NSAIDs]?’; ‘Are there side-effects?’*	[144]
	Fragmented care—lack of integrated communication among providers	*I don’* *t get the impression that there is any real discussion between them [health care providers], they just pass on information. You are just an object, a ping-pong ball going to and fro. They pass on x-rays and little notes to colleague that are sealed and you aren’* *t shown what is in them.*	[144]
	Reliance on natural supplements for cartilage health	*A friend told me: ‘this year the doctor gave me cod-liver oil’. It’s very important for the cartilage. We can also take shark cartilage as a dietary supplement. It’s my GP who told me first that Harpagophytum was relevant for arthritis. And as they are all natural products, I thought ‘why not’?*	[150]
	Belief in dietary supplements to support joint function	*I read cautiously all the things written on these products. And actually, when you have knee problems, it’s as if the joint was not well-oiled. Dietary supplements feed the cartilage, and make the joints suppler. So it’s getting better. We do have less pain (…) By taking these products, it does help my cells to renew.*	[150]
	Skepticism about supplements for cartilage protection	*All that sort of stuff [like glucosamine] is supposed to help your cartilage and protect it. But once it’s not there, it’s not going to make more of it … once it’s gone it’s gone.*	[157]
	Epistemic ambiguity in OTC therapeutics products/Uncertainty patients experience when supplement efficacy is unverified	*“The glucosamine is supposed to affect the cartilage in your knee and strengthen it, but whether it did or not, I don’t know. I just kept taking them until such a time that I decided it wasn’t doing me any good”.*	[158]
	Incorporation of “natural” supplements into individualized self-care practices/Biocultural adoption of nutraceutical regenerative logics	*“I tried chondroitin, it’s from fish. They tell you that it’s good to rebuild the cartilage of your knees”.*	[158]
	Perception of pharmacists as accessible sources of information	*I think pharmacists probably know a lot more information sometimes than the doctor does. Because they’re dealing with people at the coalface all the time.*	[22]
	Need for clear, personalized explanations	*I think it’s a good way of allowing people to receive something, but with some knowledge. Often you’ll go into a doctors surgery and you see all those booklets and all the wee pamphlets and things, but nobody explains them to you.*	[22]
	Facilitated access to care and acceptance of diagnosis	*It just saved me having to actually seek out that referral myself… it was nice to have that there, because sometimes you can be in denial about your diagnosis. You think, oh well I’m too young to think about that at the moment.*	[22]
	Contradictory medical advice	*They [doctor] said, ‘the walking’s agitating you, your joints, so stop it’.*	[142]
	Lack of exercise guidance	*I haven’t had any advice about exercising and what exercises to do.*	[142]
	Balancing activity with fear of further joint damage	*I’ve got a window of time to do all these things in. But then, at the same time, I’ve got to do this in a way that doesn’t impact that window of time, make it shorter than it otherwise would be. So it’s really, yeah, finding that balance.*	[157]
	Uncertainty about exercise effects on cartilage	*Am I strengthening it or am I sort of destroying the cartilage? I don’t know.*	[157]
	Value of education in pain management	*I learned so much from [the physiotherapist]…I learnt about pain management…it helped me understand arthritis much better.*	[147]
	Exercise as self-management	*[Exercise helps you understand] how to cope with pain…that exercise does help ease the pain and helps your mobility…but there is no cure for [arthritis], it’s learning to live with it.*	[141]
	Importance of physical activity for mood and function	*Keeps the body moving, takes your mind off it, it’s good to be outside. Yea, keeping active, or else if you’ve got osteo [osteoarthritis], it can get you right down, if you stay inside you just mope about it.*	[143]
	Viewing cycling as a low-load, beneficial activity	*[Biking] there’s no load on your knees… it’s keeping you in motion, keeping you active, and it’s not stress or anything on your knees.*	[157]
	Pain and activity limitation	*It’s hard to get going on a bike and very painful. It’s absolute agony in spite of painkillers, so any activity is very limited.*	[142]
	Fear of exercise due to persistent pain	*Not only does it hurt when you [move], but it would hurt the next day. The pain never lets you forget…and believe me, I don’t. The only thing I can do is not do it again. Avoid exercise, avoid the pain.*	[155]
	Recognition that physiotherapy becomes ineffective once degeneration reaches “bone-on-bone” contact, underscoring the gap to surgical care	*“I’ve tried physio over the years … but they’re not able to help bone on bone when it gets to that stage”.*	[158]
	Feeling brushed off by doctors and valuing genuine interest	*I’d gone to doctors and things, and you know, I’ve been brushed off. “Oh well, your knees are wearing out, tough”. So it was quite refreshing to find somebody who was actually interested.*	[22]
	Financial barriers to exercise participation	*When you go up to the pool it’s $2 and then you get charged $5 to go into the aerobics, well that’s really, sort of, you know, pay for the guy’s time, that person’s time but when you’re on a pension you haven’t got that.*	[154]
	Media-amplified therapeutic hype and expectation formation/News coverage shapes patient expectations before clinical validation	*“[The doctor] told me it was a new technique, it was just an injection. They would inject it and then the two bones would stop rubbing each other. Two weeks later it was on the news, they were saying that it got everybody walking without any problems”.*	[158]
	Regenerative hope amid biomedical exclusion/Tension between patient aspirations for cell-based therapies and clinical eligibility	*“I’ve heard people have this stuff they inject in. They take your fat cells and they grow it and they put it back in to the joint, so it’s just like a cartilage. I was hoping to get that, I would have preferred it, but the surgeon said it’s too far gone”.*	[158]
	Diagnostic imaging-driven perception of joint degradation severity	*“They’ve shown me the pictures of the inside of my knees, it is literally just two round circles—balls—with nothing on them”.*	[158]
	Regret about delaying specialist referral due to trivializing OA, resulting in missed opportunities for earlier, less invasive care	*“If I had seen the specialist early, then they could probably do an alternative treatment. But instead of going to the doctor, I thought oh well, it’s arthritis it doesn’t matter. Until I couldn’t bear the pain anymore, and then I went and found out it was too late”.*	[158]
	Frustration with physiotherapy limitations in irreversible cartilage loss, underscoring need for mechanical restoration	*“I haven’t got the cartilage there, so [the physiotherapy] can’t do much about that … They can’t replace my cartilage: I’ve got to put the cushion back into my knee”.*	[158]
	Uncertainty and apprehension about knee surgery	*We should have more information, as practitioners, to know what to do. (…) I feel it is vague for us, and also for the patients! Practitioners of my age are not so confident with surgery… it (knee OA) is a pathology that makes us feel uncomfortable. There is no problem with hips surgery but when the knee is concerned, it is a frightening surgery.*	[150]
	Dichotomous perceptions of surgical outcomes	*I don’t want knee surgery, I’ve seen it happen; I’ve seen people have it very successfully and I’ve seen it be a disaster.*	[147]
	Lack of informed consent and patient-centered care	*Public sector clinicians simply ask “do you want the surgery or not” and do not provide any written or verbal information about the surgery.*	[148]
	Emotional impact of dismissing surgical options	*They do not recommend the operation [knee arthroplasty]! They don’t recommend the operation! Straightaway physiotherapy, go and do electrical massage or injections; can you imagine I feel I am dying.*	[148]
	Age-based biases delay intervention and compound patient urgency	*“I’ve just been waiting, putting up with the pain, because all the doctors say I’m too young. But everyone in my family dies before 70. So, what, am I going to live for the rest of my life in pain? The x-rays clearly show that there’s no cartilage in my knee”.*	[158]
	Prospective trust in biomedical innovation/Forward-looking patient faith and optimism about future medical advances reshaping long-term outcomes	*“They say that it only lasts for 10 years and that’s why they try to put it off as long as possible. I think it’ll last longer … I just think with how medical things improve all the time, they will make it better and make it last longer”.*	[158]
	Desire for expertise and innovation—seeking knowledgeable and up-to-date care	*I would have preferred [clinician] to have had training for this disease. I’* *m sorry (he) does not have more time to get information and pass it on to me. Are there any new developments—with lesser side effects? (He) does not even know about it’* *. The ideal thing would be a drug that regenerates … that rebuilds damaged cartilage. Can you graft cartilage?* *Is there an efficient treatment other than operation?*	[144]
	Perceived neglect—OA research is undervalued compared to other conditions	*Is there a way to prevent it in young people? Is research progressing?* *Are there a lot of researchers working in this field? Do they have means available? Are there experimental centers? Do research labs really do proper research on this disease? What you hear a lot about is diabetes. You get the impression that in spite of all the research, OA is left out …yet commercially, it brings in a lot for doctors, chemists and labs.*	[144]

## Data Availability

No new data were created or analyzed in this study. Data sharing is not applicable to this article.

## Abbreviations

The following abbreviations (in alphabetic order) are used in this manuscript:

ACR	American College of Rheumatology
ADL	Activities of daily living
ADAMTS	A Disintegrin and Metalloproteinase with Thrombospondin Motifs
ADME(T)	Absorption, distribution, metabolism, excretion (and toxicity)
APLAR	Asia-Pacific League of Associations for Rheumatology
BSR	British Society for Rheumatology
BMAL1	Brain and muscle Arnt-like protein-1
CAGR	Compound annual growth rate
CAPA	Canadian Arthritis Patient Alliance
Clock	Circadian locomotor output cycles kaput
C_max_	Maximum plasma concentration
COMP	Cartilage oligomeric matrix protein
CRY	Cryptochrome
COX	Cyclooxygenase
CTSMA	Connective tissue structure-modifying agent
CVD	Cardiovascular disease
CYP	Cytochrome P450
DAMPs	Damage-associated molecular patterns
DMARD	Disease-modifying antirheumatic drug
DMOAD	Disease-modifying osteoarthritis drug
ECM	Extracellular matrix
ESCEO	European Society for Clinical and Economic Aspects of Osteoporosis, Osteoarthritis and Musculoskeletal Diseases
EULAR	The European Alliance of Associations for Rheumatology
FDA	Food and Drug Administration
FIP	International Pharmaceutical Federation
Fn-fs	Fibronectin fragments
GAIT	Glucosamine hydrochloride/chondroitin sulfate Arthritis Intervention Trial
GI	Gastrointestinal
IL-1β	Interleukin-1 beta
iPSC	Induced pluripotent stem cell
MetS	Metabolic syndrome
MMPs	Matrix metalloproteinases
MSC	Mesenchymal stem cell
MRI	Magnetic resonance imaging
NSAID	Nonsteroidal anti-inflammatory drug
OA	Osteoarthritis
OAAA	Osteoarthritis Action Alliance
OAFI	Osteoarthritis Foundation International
OAI	Osteoarthritis Initiative
OARSI	Osteoarthritis Research Society International
OTC	Over-the-counter
PALs	Physical activity levels
PCC	Patient-centered care
PER	Period
PICs	Pro-inflammatory cytokines
PRG4	Proteoglycan 4 (lubricin)
PRP	Platelet-rich plasma
PTOA	Post-traumatic osteoarthritis
RA	Rheumatoid arthritis
RCT	Randomized controlled trials
RNS	Reactive nitrogen species
ROS	Reactive oxygen species
SNRI	Serotonin-norepinephrine reuptake inhibitor
SODFs	Solid Oral Dosage Forms
SYRADOA	Symptomatic rapid-acting drugs for osteoarthritis
SYSADOA	Symptomatic slow-acting drugs for osteoarthritis
THA	Total hip arthroplasty
TKA	Total knee arthroplasty
TNF-α	Tumor necrosis factor-alpha
CTX-II	Type II collagen C telopeptides
Helix-II	Type II collagen helical
VAS	Visual Analog Scale
WOMAC	Western Ontario and McMaster Universities Osteoarthritis Index
